# Genomic characterization of KPC-2 and NDM coproducing carbapenem-resistant *Klebsiella pneumoniae* in a hospital: discovery of ST1869 clone and a novel hybrid plasmid

**DOI:** 10.1128/spectrum.04195-25

**Published:** 2026-08-13

**Authors:** Chengfeng Lin, Jingjie Song, Rongjiao Li, Leping Yan, Mei Yuan, Fangyin Zeng, Shihan Zeng

**Affiliations:** 1 Department of Clinical Laboratory, Southern Medical University, Fifth Affiliated Hospital 70570, Guangzhou, China; 2 School of Laboratory Medicine and Biotechnology, Southern Medical University 70570, Guangzhou, China; Newcastle University, Newcastle Upon Tyne, United Kingdom; International Center for Chemical and Biological Sciences, University of Karachi, Karachi, Pakistan

**Keywords:** *bla*_KPC_, *bla*_NDM_, KN-CRKP, plasmid, genomic features

## Abstract

To characterize the plasmid architecture and molecular background of KPC-NDM coproducing carbapenem-resistant *Klebsiella pneumoniae* (KN-CRKP) in a South China hospital. Five KN-CRKP isolates were collected, including three from one patient. All underwent Illumina sequencing; two (ST11 and ST1869) additionally had Nanopore sequencing. Antimicrobial susceptibility testing strain sequence types, conjugation assays, resistance gene profiling, plasmid typing, genetic structure comparison, core-genome single nucleotide polymorphisms (SNPs) analysis, and plasmid clustering were performed. All isolates exhibited an imipenem minimum inhibitory concentration (MIC) of ≥128 µg/mL and harbored multiple resistance genes. One isolate (1/5) belonged to ST1869 and co-harbored *bla*_KPC-2_ and *bla*_NDM-5_. The *bla*_NDM-5_-carrying plasmid was a novel IncI1/X3 fusion plasmid that also carried *bla*_CMY-42_. Unlike several IncX3 plasmids carrying blaNDM in publicly available KN-CRKP genomes from South China, this IncI1/X3 hybrid lacked a complete conjugative transfer system. ST11 was the predominant clone (4/5), co-harboring *bla*_KPC-2_ and *bla*_NDM-1_. A rare genetic structure, *Δ*IS*Kpn6-bla*_KPC-2_-IS*Kpn28*, was identified on IncFII plasmids carrying *bla*_KPC-2_. Plasmid clustering analysis of 126 comparative KN-CRKP genomes showed diverse sequence types and plasmid backgrounds associated with the KPC/NDM co-production pattern. The observed plasmid diversity and structural variation in KN-CRKP support continued genomic surveillance, with particular attention to the ST1869 clone, the novel IncI1/X3 hybrid plasmid harboring *bla*_NDM-5_ and *bla*_CMY-42_, and the rare “*Δ*IS*Kpn6-bla*_KPC-2_-IS*Kpn28*” genetic structure. Expanded genomic data on KN-CRKP are needed to further elucidate its resistance mechanisms and plasmid evolutionary trajectories.

**IMPORTANCE:**

The co-production of KPC and NDM carbapenemases in *Klebsiella pneumoniae* poses a formidable threat to clinical antimicrobial therapy, as these enzymes confer resistance to virtually all β-lactam agents, including carbapenems. Here, we report novel genomic features of KN-CRKP in South China, including the emergence of the ST1869 clone, a unique IncI1/X3 hybrid plasmid harboring blaNDM-5 and blaCMY-42, and the rare ΔISKpn6-blaKPC-2-ISKpn28 genetic structure. These findings substantially expand current understanding of plasmid evolution and resistance gene dissemination in this region. The identification of diverse resistance mechanisms and clonal backgrounds supports enhanced genomic surveillance and infection-control awareness for pan-resistant Enterobacterales.

## INTRODUCTION

The widespread use of carbapenems has driven the rapid global dissemination of carbapenem-resistant *Enterobacteriaceae* (CRE). A 2016–2018 study reported a 97.4% carbapenemase gene (CRG) positivity rate (911/935) among Chinese isolates (1), indicating that carbapenemase-mediated resistance represents a core mechanism underlying the CRE epidemic in China.

*bla*_KPC_ and *bla*_NDM_, the most prevalent carbapenemase genes in CRE, are classified as Class A and Class B enzymes in the Ambler scheme, respectively (1), and differ in their susceptibility to avibactam inhibition: KPC is inhibited, whereas NDM is not. Consequently, KN-CRKP poses a major challenge to β-lactam/β-lactamase inhibitor combinations, leaving limited therapeutic options, such as colistin and tigecycline (2). Both *bla*_KPC_ and *bla*_NDM_ are primarily carried by plasmids, insertion sequences, transposons, and other mobile genetic elements, enabling horizontal transfer, plasmid fusion, and structural rearrangement under antimicrobial selective pressure (3, 4). Among adult patients, KN-CRKP isolates account for 1.8% of CRE and have exhibited a year-on-year upward trend, increasing from 0.28% in 2016 to 0.58% in 2017 (1, 5). Reports from China and other regions have described KN-CRKP involving diverse clonal backgrounds, including ST11, ST15, ST1049, ST12, and ST3493 (5–7), underscoring the need to characterize local genomic contexts (8, 9). To elucidate local resistance mechanisms, five KN-CRKP clinical isolates (including one ST1869) were characterized and integrated with 126 publicly available genomes for comparative analysis.

## MATERIALS AND METHODS

### Isolate collection and identification

Non-duplicate carbapenem-resistant *K. pneumoniae* isolates recovered from clinical specimens at our institution in 2024 were screened by PCR, yielding five strains co-harboring *bla*_KPC_ and *bla*_NDM_, which were subsequently identified using the VITEK 2 COMPACT system. *Escherichia coli* ATCC 25922, *Klebsiella pneumoniae* ATCC BAA-1705, and ATCC BAA-2146 were used as quality control strains. *E. coli* C600 served as the recipient strain in conjugation assays. Owing to the anonymization of clinical records, metadata, including inpatient departments, wards, and detailed treatment histories, were unavailable.

### Conjugation assay

The transferability of carbapenem resistance in KN-CRKP isolates was assessed by conjugation assays using rifampicin-resistant *E. coli* C600 as the recipient strain. Donor and recipient strains were mixed at a defined ratio and co-cultured for 24 h at 37°C. Transconjugants were selected on LB agar plates supplemented with rifampicin (100 µg/mL) and imipenem (2 µg/mL). Three randomly selected colonies from each conjugation plate were subjected to PCR amplification and sequencing.

### Detection of bla_KPC_ and bla_NDM_ by PCR

*bla*_KPC_ and *bla*_NDM_ were detected by PCR with primers listed in Table S1. All amplicons were subsequently sent to Sangon Biotech (Shanghai) Co., Ltd. for Sanger sequencing. The resulting sequences were aligned against the NCBI database (http://blast.ncbi.nlm.nih.gov/Blast.cgi) using MEGA 12 (10).

### Antimicrobial susceptibility testing

Antimicrobial susceptibility profiling was initially performed using the VITEK-2 COMPACT system, which includes the following agents: amoxicillin/clavulanic acid (AMC), piperacillin/tazobactam (TZP), cefuroxime (CXM), cefuroxime axetil (CXA), cefoxitin (FOX), ceftazidime (CAZ), ceftriaxone (CRO), cefoperazone/sulbactam (CSL), cefepime (FEP), ertapenem (ETP), imipenem (IPM), amikacin (AMK), levofloxacin (LVX), and trimethoprim/sulfamethoxazole (SXT).

Susceptibility to aztreonam (ATM) and ceftazidime/avibactam (CZA) was determined separately using the Kirby-Bauer disk diffusion method. Additionally, the MIC of imipenem was determined by broth microdilution in triplicate, following CLSI guidelines. All experiments were repeated three times (11).

### Whole genome sequencing and bioinformatics analysis

Genomic DNA extracted from KN-CRKP isolates using a commercial kit (Tiangen Biotech) was sequenced on the NovaSeq X Plus platform (Novogene, China), with all five strains subjected to Illumina short-read sequencing. To obtain complete plasmid structures, representative ST11 strain 230-25 and ST1869 strain 231-20 were selected for Nanopore long-read sequencing. Following quality control (12), the raw reads were assembled using SPAdes v3.13.1 (13). The resulting genomes were annotated with Prokka v1.14.5 (14), and multilocus sequence typing (MLST) was performed using MLST v2.18.0 to determine sequence types (STs) (15). The resistome and plasmid replicon profiles were predicted through the Center for Genomic Epidemiology web server (https://cge.food.dtu.dk), with plasmid incompatibility groups further characterized using PlasmidFinder v2.1 under thresholds of ≥95% coverage and ≥85% identity (16). Contigs carrying *bla*_KPC_ and *bla*_NDM_ were identified from all Illumina-assembled genomes. Representative isolates spanning distinct STs and NDM variants were selected for long-read sequencing on the Nanopore platform (Novogene, China) to resolve plasmid structures.

### Localization and genetic structure analysis of bla_KPC_ and bla_NDM_

The localization and genetic structure of *bla*_KPC_ and *bla*_NDM_ were investigated by integrating whole-genome sequencing data from the study isolates with publicly available sequences from NCBI. Circular plasmid comparisons were generated using BRIG v0.95 (17), and homologous plasmids along with their genetic contexts were identified via BLASTn. Genetic structure comparisons were visualized with BLASTN 2.16.0+ (18), Easyfig 2.2.5 (19), and esthetically refined in Adobe Illustrator. Core-genome SNP analysis was performed with Snippy v4.6.0 (20) (minimum sequencing depth ≥10×, base quality ≥20, and allele frequency ≥75%) to assess genetic relatedness between the isolates and selected publicly available strains from South China. Parameter settings followed established standards for bacterial genome comparison, using default quality thresholds (21). Conjugative transfer modules, including the origin of transfer (oriT), relaxase, type IV coupling protein (T4CP), and type IV secretion system, were predicted *in silico* using oriTfinder (22).

### Strain screening and plasmid cluster analysis

To systematically characterize plasmids, genomic assemblies were retrieved from the NCBI Pathogen Detection database (https://www.ncbi.nlm.nih.gov/pathogens/microbigge/#). Searches were performed using "*bla*_KPC_" and "*bla*_NDM_" as query terms, with the species restricted to *K. pneumoniae*, and the search date was June 20, 2025.

By comparing and screening strains carrying both resistance genes, a list of candidate KN-CRKP isolates was obtained. High-quality genomes were retained based on the following criteria: (i) complete genome or clear annotation of circular plasmids; (ii) for draft genomes, contig number ≤200 and N50 ≥ 50,000 bp; and (iii) exclusion of assemblies containing only chromosomal sequences and lacking plasmid-related contigs. Assemblies that did not meet any of the above criteria or were marked as “suppressed” or “surveillance assembly” by NCBI RefSeq were considered invalid and excluded. Ultimately, 126 KN-CRKP genomes were successfully obtained, of which 103 originated from China, covering provinces and cities including Beijing, Zhejiang, Hubei, and Guangdong. Plasmid replicon types were identified with PlasmidFinder v2.1 (threshold: ≥95% coverage, ≥85% identity), and replicator annotations were manually checked for *bla*_KPC_ and *bla*_NDM_ contigs. Strain sequence types (STs) were determined using MLST v2.18.0 (15). Strain information is provided in Table S2.

Contigs ≥ 10 kb carrying *bla*_KPC_ or *bla*_NDM_ were extracted from the 126 publicly available genomes and the complete plasmid sequences of the study isolates. Panaroo v1.3.2 was used to perform pan-genomic clustering analysis on contigs carrying *bla*_KPC_ or *bla*_NDM_ that were ≥10 kb in length. Following the developers’ recommendations for accurately constructing a prokaryotic pangenome, parameters were set to --clean-mode strict with an identity threshold of ≥95% (23) and visualized using TVBOT (24). Plasmids were annotated according to replicon type, ST, geographic region, and the genetic environment classifications of *bla*_KPC_ and *bla*_NDM_ to analyze clustering and evolutionary relationships among *bla*_KPC_- or *bla*_NDM_-carrying plasmids.

## RESULTS

### Clinical origins, ST subtypes, and high resistance characteristics of five KN-CRKP strains

Five non-duplicate KN-CRKP isolates (230-25, 230-41, 230-71, 232-97, and 231-20) were collected over a period of 8–55 days. Isolates 230-25, 230-41, and 230-71, recovered from the same patient over 23 days, exhibited 0–1 core-genome SNPs, suggesting a common clonal origin; clinical outcome data were unavailable for this laboratory-based genomic surveillance. Isolate 232-97 was recovered from urine, whereas the remaining isolates were obtained from sputum. MLST revealed that isolate 231-20 belonged to ST1869, whereas the remaining isolates were assigned to ST11 lineage (Table 1)

**TABLE 1:** Microbiological characteristics of KN-CRKP and its transconjugants^*d*^

Strains and transconjugants	CRG^*a*^	MIC^*b*^ (mg·L⁻¹)													Zone diameter (mm)	
		IPM^*c*^	AMC	TZP	CXM	FOX	FEP	CAZ	CRO	CFP-SUL	ETP	AMK	LVX	SXT	ATM	CZA
230-25	KPC + NDM	**128^*d*^**	≥**32**	≥**128**	≥**64**	≥**64**	**16**	≥**64**	≥**64**	≥**64**	≥**8**	≥**64**	≥**8**	≤20	**6**	**17**
C23025N	NDM	**8**	≥**32**	≥**128**	≥**64**	≥**64**	**16**	≥**64**	≥**64**	≥**64**	≥**8**	≥**64**	0.5	≤20	**–^*e*^**	**–**
230-41	KPC + NDM	**128**	≥**32**	≥**128**	≥**64**	≥**64**	**16**	≥**64**	≥**64**	≥**64**	≥**8**	≥**64**	≥**8**	≤20	**6**	**15**
C23041N	NDM	**8**	≥**32**	≥**128**	≥**64**	≥**64**	2	≥**64**	≥**64**	≥**64**	0.5	4	0.5	≤20	**–**	**–**
230-71	KPC + NDM	**256**	≥**32**	≥**128**	≥**64**	≥**64**	**16**	≥**64**	≥**64**	≥**64**	≥**8**	≥**64**	≥**8**	≤20	**6**	**16**
C23071N	NDM	**8**	≥**32**	≥**128**	≥**64**	≥**64**	2	≥**64**	≥**64**	≥**64**	1	≤2	0.5	≤20	**–**	**–**
C23071K	KPC	**32**	≥**32**	≥**128**	≥**64**	≥**64**	≥**32**	≥**64**	≥**64**	≥**64**	≥**8**	≥**64**	0.5	≤20	**–**	**–**
232-97	KPC + NDM	**128**	≥**32**	≥**128**	≥**64**	≥**64**	**16**	≥**64**	≥**64**	≥**64**	≥**8**	≥**64**	≥**8**	≥**320**	**6**	**15**
C23297N	NDM	**8**	≥**32**	≥**128**	≥**64**	≥**64**	**16**	≥**64**	≥**64**	≥**64**	≥**8**	≤2	0.5	≤20	**–**	**–**
231-20	KPC + NDM	**256**	≥**32**	≥**128**	≥**64**	≥**64**	**16**	≥**64**	≥**64**	≥**64**	≥**8**	≥**64**	≥**8**	≥**320**	**6**	**14**
C600	*–*	≤0.25	4	≤4	8	≤4	≤0.12	≤0.12	≤0.25	≤8	≤0.12	≤2	0.25	≤20	**–**	**–**

^*a*^ Carbapenem resistance genes, determined by PCR.

^*b*^ Minimum inhibitory concentration.

^*c*^ Amoxicillin-clavulanic acid (AMC), Piperacillin-tazobactam (TZP), Cefuroxime axetil (CXM), Cefoxitin (FOX), Ceftazidime (CAZ), Ceftriaxone (CRO), Cefepime (FEP), Cefoperazone-sulbactam (CFP-SUL), Ertapenem (ETP), Imipenem (IPM), Amikacin (AMK), Levofloxacin (LVX), Trimethoprim-sulfamethoxazole (SXT), Aztreonam (ATM), and Ceftazidime-avibactam (CZA).

^*d*^ Bold indicates drug resistance.

^*e*^ –, not tested.

### The carbapenemase gene carried by the transconjugants

Transconjugants were successfully obtained for all donor isolates except 231-20. For each conjugation experiment, three randomly selected colonies from the double-antibiotic plates were screened. Among all transconjugants screened, only *bla*_NDM_ was recovered; the single exception, strain C23071K, yielded *bla*_KPC_ alone. Table 1 summarizes the resistance profiles of all isolates and transconjugants. Despite reduced imipenem MICs (8–32 μg/mL) and retained susceptibility to FEP and ETP in *bla*_NDM_ transconjugants, transferred plasmids conferred resistance to most β-lactam agents.

### Discovery of the novel IncI1/X3 fusion plasmid and the rare bla_KPC-2_ genetic structure

Genomic analysis revealed that all KN-CRKP isolates carried a diverse repertoire of antimicrobial resistance genes (Table 2). All ST11 isolates co-harbored *bla*_NDM-1_ and *bla*_KPC-2_, whereas the ST1869 isolate carried *bla*_NDM-5_ alongside *bla*_KPC-2_. All isolates possessed IncFII-type plasmid replicons, with strain 231-20 harboring as many as six distinct replicon types.

**TABLE 2:** Resistance genes and plasmid replicons carried by isolates

Isolates	Resistance gene	Plasmid replicon^*a*^
230-25	*bla*_NDM-1_, *bla*_KPC-2_, *bla*_SHV-12_, *qnrS1*, *bla*_LAP-2_, *fosA6*, *ble*_MBL_, *sul1, sul2*, *tet(A*), *bla*_TEM-1_, *aadA2, rmtB*, *bla*_SHV-187_	IncFIB(2), IncFII(3), IncH1B
230-41	*bla*_NDM-1_, *bla*_KPC-2_, *bla*_SHV-12_, *qnrS1*, *bla*_LAP-2_, *fosA6*, *ble*_MBL_, *sul1, sul2*, *tet(A*), *bla*_TEM-1_, *aadA2, rmtB*	IncFIB(2), IncFII(3), IncH1B
230-71	*bla*_NDM-1_, *bla*_KPC-2_, *bla*_SHV-12_, *qnrS1*, *bla*_LAP-2_, *fosA6*, *ble*_MBL_, *sul1, sul2*, *tet(A*), *bla*_TEM-1_, *aadA2, rmtB*	IncFI3B(2), IncFII(3), IncH1B
231-20	*bla*_NDM-5_, *bla*_KPC-2_, *bla*_SHV-187_, *qnrS1*, *bla*_LAP-2_, *fosA6*, *ble*_MBL_, *sul2*, *tet(A*), *bla*_TEM-1_, *dfrA14*, *rmtB*, *bla*_CTX-M-65_, *bla*_CMY-42_*, emr(B*)	IncFIB, IncFII(2), IncH1B, IncR, IncX, IncI1
232-97	*bla*_NDM-1_, *bla*_KPC-2_, *bla*_SHV-12_, *qnrS1*, *bla*_LAP-2_, *fosA6*, *ble*_MBL_, *sul2*, *tet(A*), *bla*_TEM-1_, *dfrA14, rmtB, bla*_CTX-M-65_, *catA2*	IncFIB, IncFII(3), IncH1B, IncR, IncN

^*a*^ The numbers in parentheses indicate the number of plasmid replicons.

**Fig 1:**
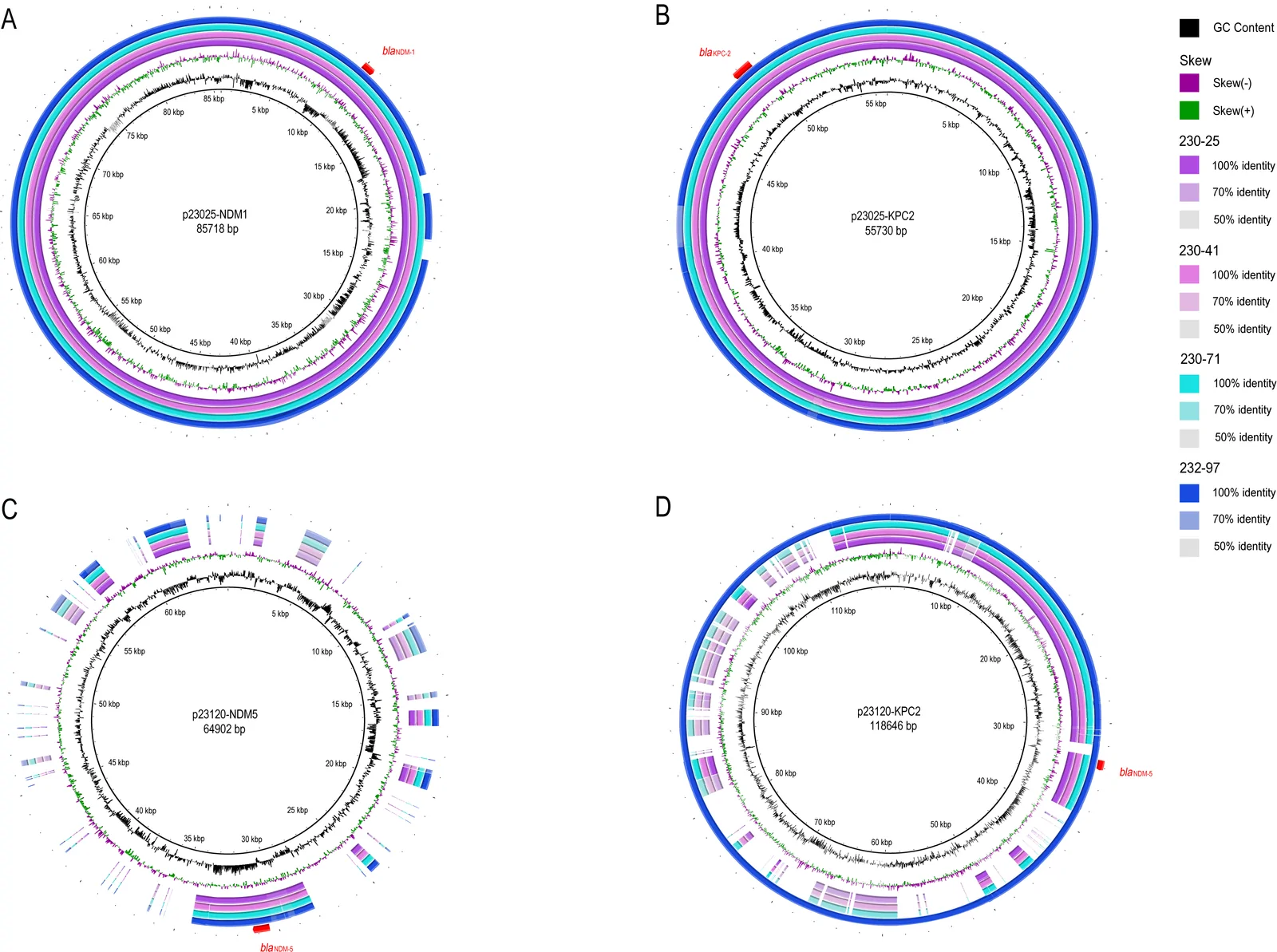
Comparison of the backbone of KN-CRKP carbapenem-resistant plasmids. (**A**) Genetic comparison of plasmids carrying *bla*_NDM-1_ in 230-25 with other isolates. (**B**) Genetic comparison of plasmids carrying *bla*_KPC-2_ in 230-25 with other isolates. (**C**) Genetic comparison of plasmids carrying *bla*_NDM-5_ in 231-20 with other isolates. (**D**) Genetic comparison of plasmids carrying *bla*_KPC-2_ in 231-20 with other isolates.

Representative strains 230-25 and 231-20 were selected for long-read sequencing based on conjugation assay results and NGS data to resolve their respective resistance plasmid architectures. In 230-25, *bla*_NDM-1_ and *bla*_KPC-2_ resided on two distinct IncFII-type plasmids (p23025-NDM1 and p23025-KPC2, respectively), whereas in 231-20, *bla*_KPC-2_ was harbored by an IncFII/R plasmid (p23120-KPC2) and *bla*_NDM-5_ by a novel IncI1/X3 hybrid plasmid (p23120-NDM5) (Table S3). These complete sequences served as references for BRIG-based comparative genomic analysis (Fig. 1), facilitating visualization of carbapenemase gene contexts and plasmid structural features.

*bla*_KPC-2_ and *bla*_NDM-1_ in isolates 230-41 and 230-71 likely resided on plasmids homologous to those in 230-25, albeit with localized regions exhibiting <100% sequence identity, suggestive of plasmid evolution throughout the sampling period. Despite acquisition of *bla*_NDM_-bearing plasmids by transconjugants C23041N and C23071N, both retained susceptibility to FEP, ETP, and AMK. This genotype-phenotype discrepancy may reflect host-plasmid interactions or incomplete resistance gene expression following horizontal transfer.

High sequence similarity between isolate 232-97 and the IncFII plasmid p23025-NDM1 indicated an IncFII-type location for its *bla*_NDM_ gene. However, BLASTN analysis (88.97% coverage with p23025-KPC2; 100% with p23120-KPC2) precluded definitive assignment of the *bla*_KPC-2_-harboring plasmid based exclusively on short-read data, with sequence variation within the 41–43 kbp region of p23025-KPC2, primarily involving the Tn3 family transposase TnAs1, indicative of a role in plasmid rearrangement.

Following BLASTn analysis of plasmids p23025-KPC2, p23120-KPC2, p23025-NDM1, and p23120-NDM5 against the NCBI nucleotide database, complete plasmid sequences with the highest query coverage were retrieved and systematically compared (Fig. S1). p23120-KPC2, p23025-KPC2, and p23025-NDM1 all detected a large number of similar plasmids, with sequence identity usually above 98%, but significant coverage differences (about 10%–90%), suggesting that these plasmids may have undergone modular recombination, fragment gain or loss. p23120-NDM5 was identified as an IncI1/X3 hybrid plasmid carrying both *bla*_NDM-5_ and *bla*_CMY-42_, whereas its closest homologous plasmid, pKC263-4, lacks *bla*_CMY-42_. An IS*Ecp1* element was identified adjacent to *bla*_CMY-42_, consistent with its established role in mobilizing this resistance gene. BLAST results showed that aside from pKC263-4 (82% coverage), all other homologous plasmids exhibited only 42% coverage, as they represent typical IncX3-type plasmids rather than the hybrid structure of p23120-NDM5. These findings collectively suggest that p23120-NDM5 may represent a novel multidrug-resistant plasmid architecture.

### Plasmid clustering analysis of 126 KN-CRKP strains

**Fig 2:**
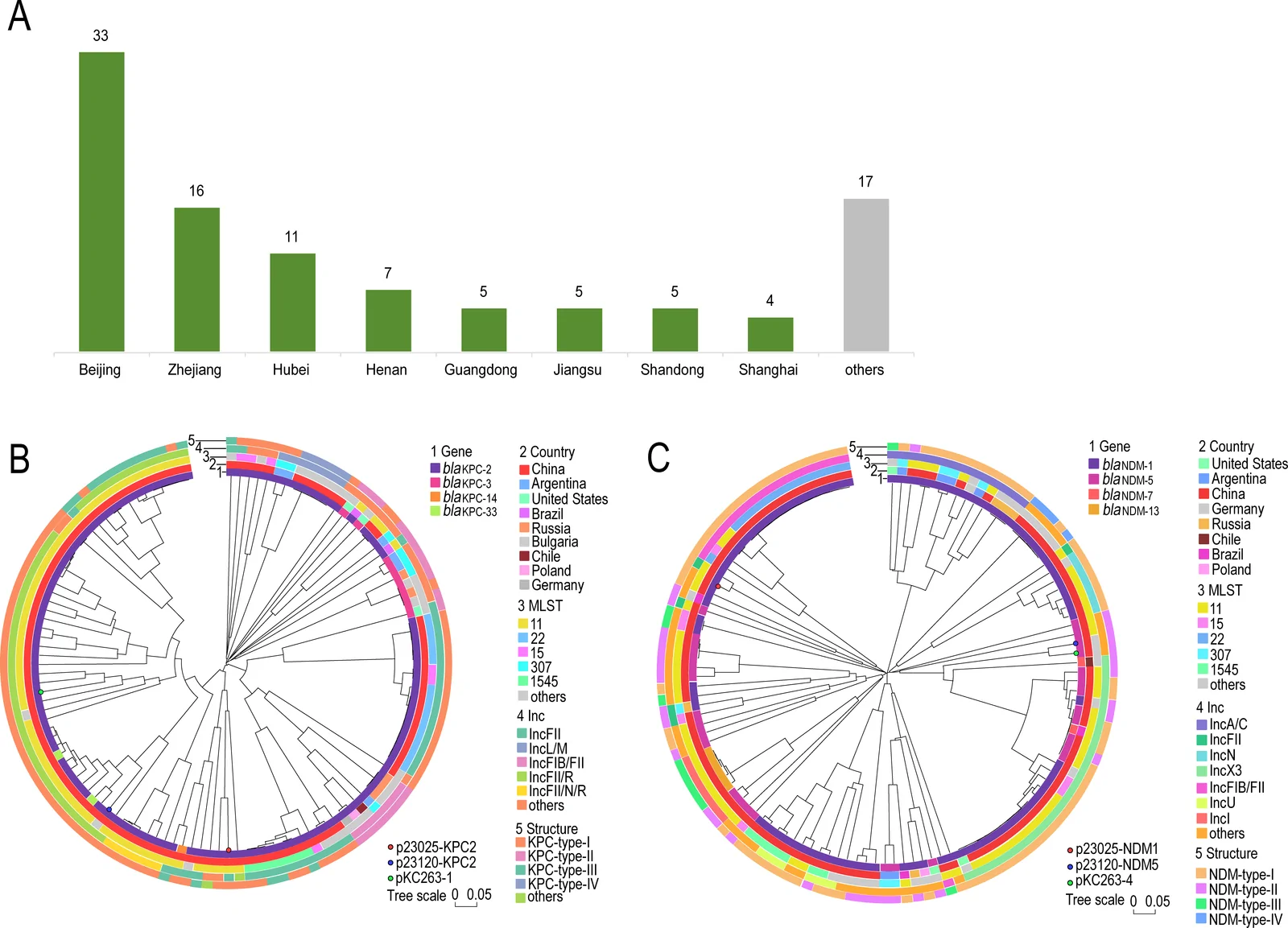
Distribution and plasmid clustering analysis of KN-CRKP. (**A**) Regional distribution of KN-CRKP in China. (**B**) Clustering analysis of plasmids carrying *bla*_KPC_. (**C**) Cluster analysis of plasmids carrying *bla*_NDM_.

To further elucidate KN-CRKP genomic features, 126 publicly available genomic data sets were combined with plasmid sequences from isolates 230-25 and 231-20. Following exclusion of chromosomal *bla*_KPC_ and *bla*_NDM_ loci, pan-genomic clustering and statistical analyses were performed (strain details in Table S2). These 126 isolates originated from nine countries, predominantly China (*n* = 103), with available data concentrated in Beijing, Zhejiang, and Hubei and limited representation from Guangdong and other provinces (Fig. 2A). Sputum (28, 22.2%) and blood (18, 14.2%) were the predominant specimen sources among 16 types identified, followed by urine (10, 7.9%). MLST analysis resolved 23 STs, with ST11 predominating (*n* = 62), followed by ST22 (*n* = 14), ST307 (*n* = 7), and ST1545 (*n* = 7).

*bla*_KPC_ variants varied geographically, with *bla*_KPC-2_ predominating in China. Among 122 *bla*_KPC_-harboring plasmid types, IncFII/R was the most prevalent (45, 36.9%), including p23120-KPC2 (Fig. 2B). Of 122 *bla*_NDM_-bearing plasmids, IncX3 predominated (26, 21.3%), followed by IncI1 (7, 5.7%); the IncI1/X3 hybrid was identified exclusively in pKC263-4 and p23120-NDM5, which clustered on the same branch (Fig. 2C), suggestive of a common hybrid origin. Notably, a 2025 wastewater isolate from Luzhou, Sichuan, harbored *bla*_KPC_ and *bla*_NDM_ on IncFII and IncU plasmids, respectively, both common in clinical settings.

### Genetic environment of bla_KPC_ and bla_NDM_ in KN-CRKP

**Fig 3:**
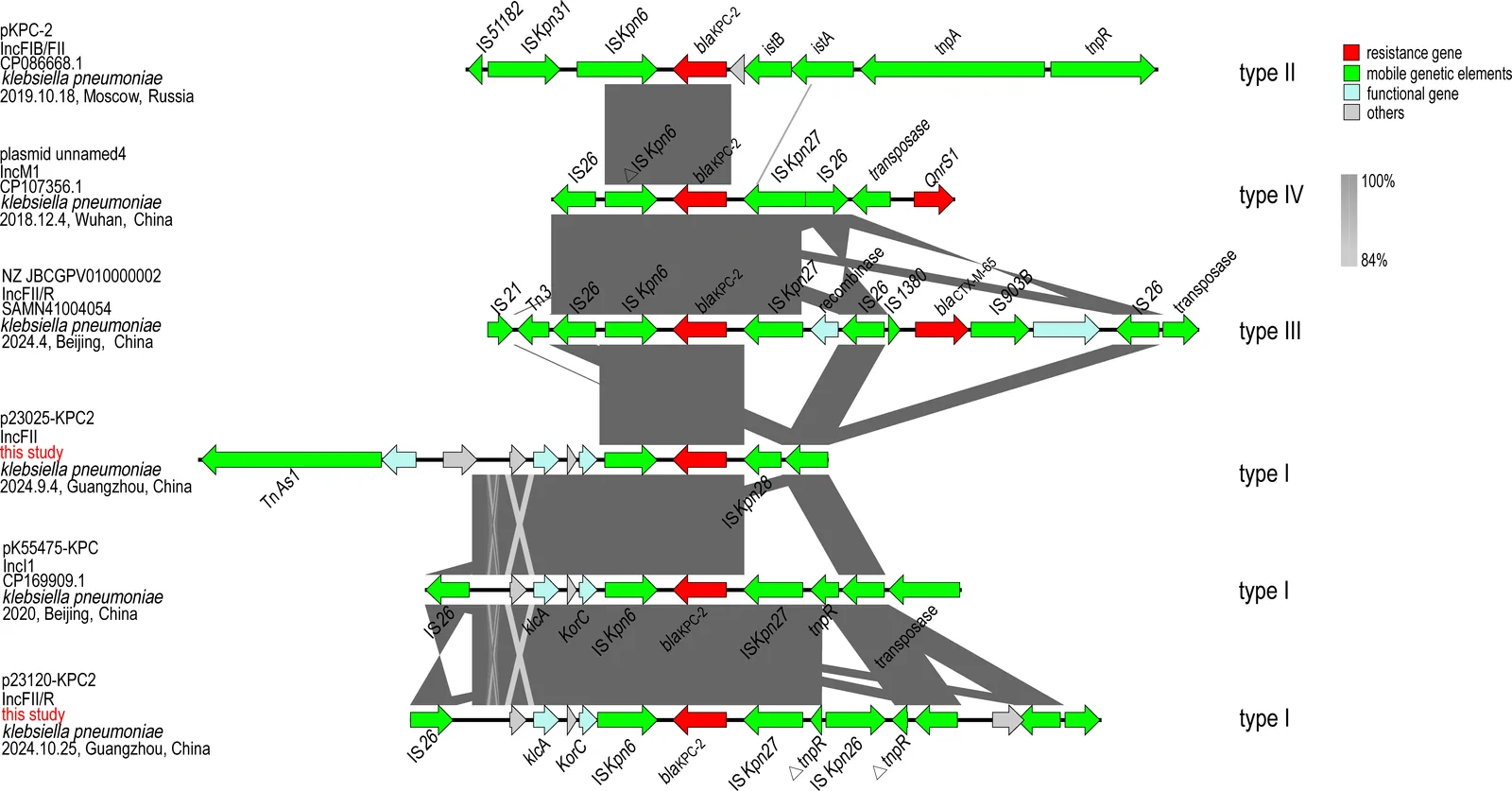
KN-CRKP *bla*_KPC_ genetic structure. Open reading frames are indicated by arrows. Red, resistance genes; green, mobile genetic elements; blue, functional protein genes; and gray, other genes.

Genetic structure analysis of 126 KN-CRKP strains from NCBI revealed four distinct structural arrangements flanking the *bla*_KPC-2_ gene (Fig. 3): KPC-type-I (*n* = 82): Tn*3*-associated core structure: “*hp-klcA-hp-KorC-Δ*IS*Kpn6-bla*_KPC-2_-IS*Kpn27-tnpR*”; KPC-type-II (*n* = 19): Tn*4401* derived “IS*Kpn6- bla*_KPC-2_-*istB-istA-tnpA-tnpR*”; KPC-type-III (*n* = 18): “IS*21*-Tn*3*-IS*26-Δ*IS*Kpn26-bla*_KPC-2_-IS*Kpn27-recombinase*-IS*26*”; and KPC-type-IV (*n* = 5): “IS*26*-IS*Kpn6-bla*_KPC-2_-IS*Kpn27*-IS*26*-IS*26*-transposase-*QnrS1.*” Representative examples are provided in Fig. S2. The *bla*_KPC_ genetic environment in isolates 230-25 and 231-20 corresponded to KPC-type-I. Notably, p23025-KPC2 harbored the rare “*Δ*IS*Kpn6-bla*_KPC-2_-IS*Kpn28*” structure, rather than the common IS*Kpn27* flanking, suggestive of a distinct recombination event within this KPC-2 transfer module.

Analysis of the *bla*_NDM_ flanking regions identified four distinct genetic structures (Fig. S3): NDM-type-I (*n* = 81) core structure is “*bla*_NDM-1_-*ble-trpF-dsbD-cutA-groS-groL-IS91*”; NDM-type-II(*n* = 31) is “*bla*_NDM-1_-*ble-trpF-dsbD*”; NDM-type-III(*n* = 10) is “*bla*_NDM-1_-*ble-trpF*”; and NDM-type-IV (*n* = 4) is “IS*Kpn19*-IS*3000-bla*_NDM-1_-IS*Kpn19-Rec-ORF3*-Tn*3*-IS*26.*” Representative genetic maps for each type are provided in Fig. S4. *bla*_NDM-1_ in strains 230-25 corresponded to NDM-type-I, whereas *bla*_NDM-5_ in 231-20 corresponded to NDM-type-II. The principal inter-type variation resided in the accessory gene repertoire flanking *bla*_NDM_.

### SNP analysis suggests that ST1869 is highly homologous to Guangzhou KC263

**Fig 4:**
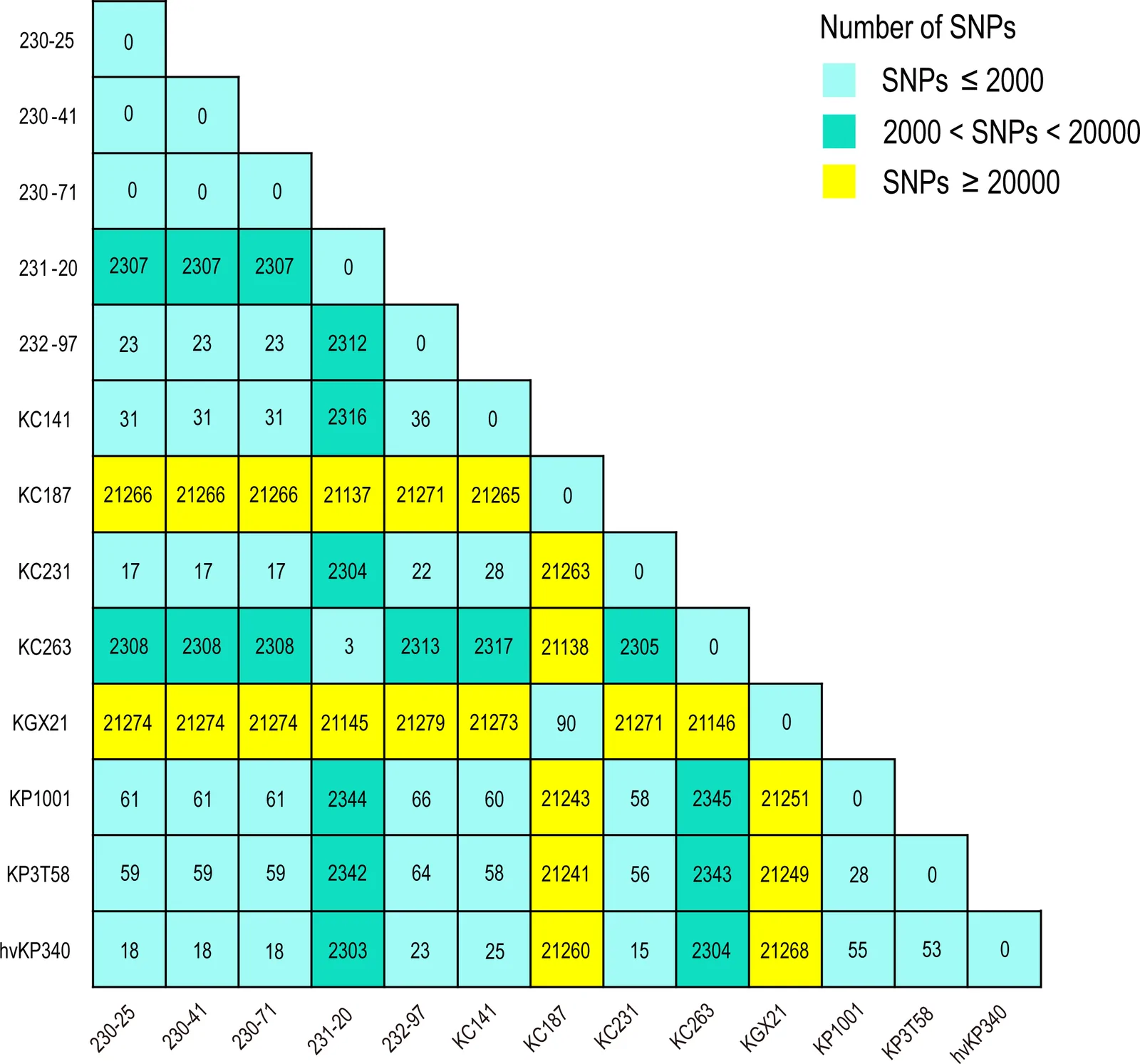
KN-CRKP paired SNP analysis matrix in South China.

From 126 publicly available KN-CRKP genomes retrieved from NCBI, eight isolates originating from Southern China were selected for core-genome SNP analysis. Strain 230-25 served as the reference, with sequencing data from five additional target strains included for comparative analysis (Fig. 4). Pairwise SNP comparisons among 230-25, 230-41, and 230-71 revealed 0–1 SNP differences, consistent with a clonal origin, while remaining ST11 isolates diverged by fewer than 100 SNPs from the reference.

Strain details, MLST types, and carbapenemase-resistant plasmid profiles are provided in Table S2. Isolates KC141, KC187, KC231, and KC263 were all recovered from Guangzhou; notably, KC263 and isolate 231-20 shared ST1869, with only three core-genome SNPs separating them, suggestive of a common clonal origin or shared local transmission. Comparative analysis of plasmids p23025-KPC2, p23120-KPC2, p23025-NDM1, and p23120-NDM5 with other KN-CRKP carbapenem-resistant plasmids from Southern China revealed that these vectors generally harbor numerous mobile genetic elements. p23120-KPC2 harbored additional ion-metabolism-related genes absent from the reference plasmid p6-hvKP340, suggestive of IncR-mediated plasmid fusion. Similarly, p23025-KPC2 carried *bla*_SHV-12_, not present in pKP3T58-KPC, presumably mobilized by flanking transposable elements (Tn*As1*, IS*48*, and IS*26*) (Fig. S5).

While both IncX3 and IncI1 plasmids carrying *bla*_NDM_ typically encode intact conjugation systems, the hybrid IncI1/X3 plasmid lacks intact conjugation systems. In contrast, p23025-NDM1 possesses a relatively complete conjugation system, which may explain the higher frequency of NDM-positive transconjugants observed for this plasmid (Fig. S6). Although p23025-KPC2 has lost most of its conjugative transfer proteins, oriTfinder analysis identified a 470-bp oriT sequence on this plasmid, which shares 100% identity with the corresponding region in the mobilizable plasmid pC2414-2-KPC (CP039820.1). This finding suggests that p23025-KPC2 retains the potential for horizontal transfer, though its transfer efficiency is expected to be substantially lower than that of p23025-NDM1. The conjugative transfer systems of p23120-KPC2 and p23120-NDM5 are incomplete, which may explain the failure of conjugation in 231-20.

## DISCUSSION

Five KN-CRKP isolates were recovered over a 2-month period at the Fifth Affiliated Hospital of Southern Medical University. The principal findings included: (i) an ST1869 isolate differing from ST11 solely in the *phoE* allele and highly homologous to the publicly available Guangzhou strain KC263; (ii) a novel IncI1/X3 hybrid plasmid harboring *bla*_NDM-5_ and *bla*_CMY-42_; and (iii) the rare *Δ*IS*Kpn6-bla*_KPC-2_-IS*Kpn28* genetic structure flanking *bla*_KPC-2_. These findings indicate close genomic relatedness among serial isolates, plasmid recombination, and diversification of resistance modules among local KN-CRKP.

Although ST11 and ST258 represent the predominant CRKP clones globally (25, 26), sporadic reports of additional STs, including ST1869 (isolate 231-20), have emerged. Among the seven MLST alleles distinguishing ST11 from ST1869, only *phoE* (outer membrane phosphoporin E) exhibited variation, suggestive of an ST11-related lineage. *phoE* mediates outer membrane phosphorus diffusion (27). This locus is inherently mutable, driving novel ST emergence (28, 29). ST11 strains harbored *phoE*1, whereas ST1869 carried *phoE*9. Although *phoE* mutations have been implicated in carbapenem resistance (30, 31), evidence remains insufficient to confirm whether the phoE1-to-phoE9 transition modulates resistance, virulence, or environmental adaptability. Notably, isolate 231-20 differed from Guangzhou KC263 by merely three core-genome SNPs; both shared ST1869, indicating close genomic relatedness within the available Guangzhou isolates and warranting inclusion in routine genomic surveillance.

p23120-NDM5, an IncI1/X3 hybrid harboring *bla*_NDM-5_ and *bla*_CMY-42_, lacked a complete conjugative apparatus. IncI1 plasmids typically carry *ampC* and occur in food animals and clinical *Enterobacteriaceae* (32). IncX3 plasmids are key *bla*_NDM_ vectors. A *NikA-NikB-trbC-trbB-trbA-bla*_CMY-42_ region differentiated p23120-NDM5 from pKC263-4, with IS*Ecp1* adjacency suggestive of mobile element-mediated acquisition. Substantial fragment deletions relative to pKP0937-2 and pKGX21-NDM-including near-complete loss of conjugative genes-were consistent with plasmid fusion and possible effects on plasmid maintenance (33, 34). *bla*_NDM_-harboring plasmids predominated within IncX3 and IncI1 groups, both common in available data sets and potentially underpinning hybrid emergence. The 126 genomes originated from diverse contexts with discontinuous sampling, serving as comparative genomics resources rather than epidemiological bases.

IS*Kpn28*, rather than IS*Kpn27*, was identified immediately upstream of *bla*_KPC-2_ in isolate 230-25. IS*Kpn28* mediates virulence transfer and chromosomal integration (35), exemplified by IS*Kpn28-iroBCDEN*-IS*3* toxic plasmid formation (36), and disrupts *mgrB* to confer polymyxin resistance (37). The rare *Δ*IS*Kpn6-bla*_KPC-2_-IS*Kpn28* structure, reported sporadically and most recently in Ganzhou and Ningbo (38, 39), was corroborated in 230-25, indicating national dissemination across diverse plasmid backgrounds reported in China. Given IS*Kpn28* associations with virulence and polymyxin resistance, co-transfer of resistance, virulence, and fitness loci warrants heightened surveillance.

These findings illuminate the local plasmid backgrounds and genetic environments that underlie the KPC-2/NDM-1 and KPC-2/NDM-5 co-production patterns. The KPC-2/NDM-1 combination is predominantly associated with the ST11 clone, whereas KPC-2/NDM-5 emerged in the ST1869 background, with *bla*_NDM-5_ harbored by a novel IncI1/X3 fusion plasmid. The apparent linkage between these two resistance gene combinations and specific sequence types suggests that distinct clonal backgrounds may shape the integration patterns and genetic contexts of acquired resistance determinants. Furthermore, the identification of IS*Kpn28* upstream of *bla*_KPC-2_ adds another layer of structural complexity to this combination, reflecting ongoing plasmid rearrangement and evolutionary diversification among local strains.

The conjugation experimental results were largely consistent with plasmid structural analysis: plasmids harboring *bla*_NDM_ readily yielded transconjugants, whereas p23120-NDM5 and p23120-KPC2 failed to produce detectable transconjugants due to their incomplete conjugative transfer systems. While carriage of an oriT sequence is necessary for conjugation, it is insufficient on its own; the absence of a complete transfer apparatus precludes conjugation initiation. Lower imipenem MICs in transconjugants relative to donor strains indicate that high-level carbapenem resistance in parental isolates is multifactorial, arising from the synergistic effects of co-harbored resistance genes, plasmid copy number, expression regulation, and host genetic background. Notably, some *bla*_NDM_-positive transconjugants remained susceptible to cefepime or ertapenem, suggesting that resistance gene carriage alone does not fully predict phenotype after transfer into *E. coli* C600. Potential explanations include recipient-host effects, differences in promoter activity or plasmid copy number, and uncharacterized structural changes during plasmid transfer; however, whole-genome sequencing of transconjugants was not performed; hence, these mechanisms remain hypotheses (33, 40, 41). Therefore, interpretation must integrate susceptibility testing, plasmid architecture, and gene expression data.

### Limitations

This study has several limitations. First, the sample size was small (*n* = 5), and three strains were isolated from the same patient, thereby limiting the generalizability of our findings to broader populations and geographic regions. Second, conjugation experiments were performed under laboratory conditions with limited selective pressure. The lack of whole-genome sequencing data for the transconjugants precluded comprehensive characterization of structural variations or adaptive mutations that may have arisen during plasmid transfer. Third, the long-term stability of the novel IncI1/X3 fusion plasmid and its dynamic evolution within the host remain to be evaluated. Fourth, because this study relied on retrospectively collected and anonymized clinical records, detailed treatment regimens and patient prognostic data were unavailable. Future prospective, multicenter studies integrating clinical and genomic surveillance are therefore warranted. In addition, given recent reports of KN-CRKP in wastewater, future molecular monitoring that combines clinical and environmental specimens may further elucidate the genetic diversity of these plasmids.

### Conclusion

In summary, this study identified the ST1869 KN-CRKP clone at a hospital in South China and characterized a novel IncI1/X3 fusion plasmid harboring *bla*_NDM-5_ and *bla*_CMY-42_, as well as the uncommon *Δ*IS*Kpn6-bla*_KPC-2_-IS*Kpn28* genetic structure. These findings expand the genomic landscape of KN-CRKP and provide a critical basis for local clinical prevention and control strategies. Enhanced surveillance of the ST1869 clone, IncI1/X3 plasmids harboring *bla*_NDM-5_ and *bla*_CMY-42_, and novel transposon modules is recommended to expand local molecular data sets and strengthen infection control measures.

## Supplementary Material

Reviewer comments

## Data Availability

Whole-genome sequencing data reported herein have been deposited under NCBI BioProject PRJNA1359551. Complete hybrid assemblies of strains 230-25 and 231-20, generated using Illumina short-read and Nanopore long-read sequencing, correspond to accession numbers SAMN53171962 and SAMN53172020, respectively. Draft assemblies of strains 230-41, 230-71, and 232-97, generated using Illumina short-read sequencing alone, are available under accession numbers SAMN53628860, SAMN53628922, and SAMN53629510, respectively. The following accession numbers are publicly available: SAMN53172020, SAMN53628860, SAMN53628922, and SAMN53629510. The complete hybrid assembly of strain 230-25 (BioSample SAMN53171962, GenBank accession JCAFXG000000000) has now been released and is publicly available. All sequencing data generated in this study are publicly available.

## SUPPLEMENTAL MATERIAL

Supplemental figures spectrum.04195-25-s0001.docx Legend for Figures S1 to S6.

Figure S1 spectrum.04195-25-s0002.tif Comparison of KPC-2- and NDM-carrying plasmids with related plasmids

Figure S2 spectrum.04195-25-s0003.tif Representative genetic environments of blaKPC-2 in KPC-NDM (Type I-IV).

Figure S3 spectrum.04195-25-s0004.tif KN-CRKP blaNDM Genetic structure.

Figure S4 spectrum.04195-25-s0005.tif Representative genetic environments of blaNDM in KN-CRKP (Type I-IV).

Figure S5 spectrum.04195-25-s0006.tif Comparison of p23025-KPC2, p23120-KPC2 and KN-CRKP blaKPC positive plasmids in South China.

Figure S6 spectrum.04195-25-s0007.tif Comparison of p23025-NDM1, p23120-NDM5 and KN-CRKP blaNDM positive plasmids in South China.

Supplemental tables spectrum.04195-25-s0008.docx Tables S1 to S3.

OPEN PEER REVIEW reviewer-comments.pdf An accounting of the reviewer comments and feedback.

ASM does not own the copyrights to Supplemental Material that may be linked to, or accessed through, an article. The authors have granted ASM a non-exclusive, world-wide license to publish the Supplemental Material files. Please contact the corresponding author directly for reuse.
