# Supplementary material for: Genomic characterization of KPC-2 and NDM coproducing carbapenem-resistant Klebsiella pneumoniae in a hospital: discovery of ST1869 clone and a novel hybrid plasmid
Source: Microbiol Spectr. 2026 Aug 13;14(9):e04195-25. doi: 10.1128/spectrum.04195-25 (PMC13532327; doi:10.1128/spectrum.04195-25)
Supplement: Supplementary file 1 [file reviewer-comments.pdf]

AMERICAN SOCIETY FOR MICROBIOLOGY | Microbiology Spectrum

Genomic characterization of KPC-2 and NDM coproducing carbapenem-resistant Klebsiella pneumoniae in a hospital: Discovery of ST1869 clone and a novel hybrid plasmid

Chengfeng Lin, Jingjie Song, Rongjiao Li, Leping Yan, Mei Yuan, Fangyin Zeng, and Shihan Zeng

Corresponding Author(s): Shihan Zeng, Department of Clinical Laboratory, Fifth Affiliated Hospital, Southern Medical University

**Review Timeline:**		
	Submission Date:	January 7, 2026
	Editorial Decision:	April 9, 2026
	Revision Received:	June 3, 2026
	Accepted:	June 8, 2026

Editor: Matt Bawn

Reviewer(s): Disclosure of reviewer identity is with reference to reviewer comments included in decision letter(s). The following individuals involved in review of your submission have agreed to reveal their identity: Ayaz Ahmed (Reviewer #2)

Transaction Report:

(Note: With the exception of the correction of typographical or spelling errors that could be a source of ambiguity, letters and reports are not edited. The original formatting of letters and referee reports may not be reflected in this compilation.)

DOI: https://doi.org/10.1128/spectrum.04195-25

Re: Spectrum04195-25 (Characterization of KPC- and NDM-coproducing carbapenem-resistant *Klebsiella pneumoniae* Strains and Plasmids in South China: Analysis of Epidemic Trends)

Dear Dr. Shihan Zeng:

Thank you for the privilege of reviewing your work. Below you will find my comments, instructions from the Spectrum editorial office, and the reviewer comments.

Please be aware that I reviewed this manuscript after other reviewers declined the invitation and to ensure at least two independent reviews were received.

Please return the manuscript within 60 days; if you cannot complete the modification within this time period, please contact me. If you do not wish to modify the manuscript and prefer to submit it to another journal, notify me immediately so that the manuscript may be formally withdrawn from consideration by Spectrum.

Revision Guidelines
To submit your modified manuscript, log into the submission site at https://spectrum.msubmit.net/cgi-bin/main.plex. Go to Author Tasks and click the appropriate manuscript title to begin. The information you entered when you first submitted the paper will be displayed; update this as necessary. Note the following requirements:

• Upload point-by-point responses to the issues raised by the reviewers in a file named "Response to Reviewers," NOT in your cover letter.
• Upload a compare copy of the manuscript (without figures) as a "Marked-Up Manuscript" file.
• Upload a clean .DOC/.DOCX version of the revised manuscript and remove the previous version.
• Each figure must be uploaded as a separate, editable, high-resolution file (TIFF or EPS preferred), and any multipanel figures must be assembled into one file.
• Any supplemental material intended for posting by ASM should be uploaded with their legends separate from the main manuscript. You can combine all supplemental material into one file (preferred) or split it into a maximum of 10 files with all associated legends included.

For complete guidelines on revision requirements, see our Submission and Review Process webpage. Submission of a paper that does not conform to guidelines may delay acceptance of your manuscript.

Data availability: ASM policy requires that data be available to the public upon online posting of the article, so please verify all links to sequence records, if present, and make sure that each number retrieves the full record of the data. If a new accession number is not linked or a link is broken, provide Spectrum production staff with the correct URL for the record. If the accession numbers for new data are not publicly accessible before the expected online posting of the article, publication may be delayed; please contact production staff (Spectrum@asmusa.org) immediately with the expected release date.

Publication Fees: For information on publication fees and which article types are subject to charges, visit our website. If your manuscript is accepted for publication and any fees apply, you will be contacted separately about payment during the production process; please follow the instructions in that e-mail. Arrangements for payment must be made before your article is published.

ASM Membership: Corresponding authors may join or renew ASM membership to obtain discounts on publication fees. Need to upgrade your membership level? Please contact Customer Service at Service@asmusa.org.

The ASM Journals program strives for constant improvement in our submission and publication process. Please tell us how we can improve your experience by taking this quick Author Survey.

Thank you for submitting your paper to Spectrum.

Sincerely,
Matt Bawn
Editor
Microbiology Spectrum

Reviewer #2 (Comments for the Author):

This study addresses the clinical problem related to a significant genomic epidemiology investigation of the emerging threat posed by dual-carbapenemase-producing Klebsiella pneumoniae (KN-CRKP) in South China. It provides significant information with comprehensive genomic and phenotypic data. However, a few comments need to be addressed:

• What is the rationale behind selecting only five clinical isolates from a single hospital? It limits generalizability.
• Shed some discussion on the presence of strains that showed susceptibility besides carrying resistance genes (blaNDM).
• For strain 231-20. No transconjugants were obtained, besides detecting oriT sequence. What are the reasons?
• Provide an improved method to analyze parameters such as SNA Calling thresholds, plasmid clustering, etc.
• Is there any evidence showed the single allele (phoE allele in ST1869 or its absence in ST11) variant influence pathogenicity, virulence, environmental fitness or resistance?
• Including patient therapeutic outcome will further strengthen the manuscript.
• Add Future direction, in terms of waste water surveillance or clinical surveillance.
• Add limitation of the study.

Reviewer #3 (Comments for the Author):

This manuscript addresses an important topic and contains potentially useful observations, particularly the ST1869 isolate and the putatively novel IncI1/X3 plasmid carrying blaNDM-5 and blaCMY-42. However, the manuscript currently overstates its epidemiological conclusions, the bioinformatics analysis is relatively limited for a study framed as genomic epidemiology, and several methodological and interpretive inconsistencies need to be resolved.

Major comments

1. The bioinformatics analysis is relatively limited for a study making genomic epidemiology claims. The manuscript does not include a bacterial whole-genome phylogenetic analysis that is sufficiently informative for epidemiology. The chromosomal analysis appears limited to a small core-genome SNP comparison, while much of the study relies on MLST, plasmid typing, structural comparison, oriT prediction, and plasmid clustering. These analyses are useful descriptively, but they are not sufficient to support strong conclusions about transmission, outbreak structure, or regional dissemination.

2. The epidemiological claims are too strong for the data presented. The manuscript repeatedly refers to regional outbreaks, transmission pathways, frequent plasmid transmission, and regional prevalence, but the primary dataset consists of only five local isolates collected over a short period, three from the same patient. The manuscript would be stronger if framed primarily as a genomic characterization study with comparative context rather than as definitive evidence of epidemic trends in Southern China.

3. The sequencing and assembly strategy is unclear. The methods suggest that only representative isolates underwent long-read sequencing, while the results specifically identify 230-25 and 231-20 as long-read sequenced, yet the data availability section implies complete long-read assemblies for all five isolates. This needs to be clarified explicitly.

4. Several conclusions about plasmid dissemination and circulation go beyond the evidence presented. The manuscript often moves from plasmid similarity, oriT prediction, or limited conjugation results to stronger claims about dissemination and regional circulation. These should be presented more cautiously as hypotheses rather than direct conclusions.

5. The local epidemiological metadata are too limited to support transmission-level interpretation. Apart from specimen source and the fact that three isolates came from the same patient, there is little clinical or hospital context presented. Without clearer metadata, the genomic findings cannot be interpreted robustly in an epidemiological framework.

6. The comparative genomics workflow is not sufficiently reproducible. The manuscript states that public genomes were retrieved and filtered to 126 high-quality genomes, but the search strategy, inclusion criteria, accession list, and definition of invalid entries are not described clearly enough.

Minor comments

The manuscript would benefit from substantial language editing, as there are multiple formatting and writing issues, including inconsistent headings and typographical errors. Some speculative interpretations, including genotype-phenotype explanations and broader public health implications, should also be toned down.

Dear Editor and Reviewers,

Thank you for your letter and for the reviewers' comments concerning our manuscript entitled "Genomic characterization of KPC-2 and NDM coproducing carbapenem-resistant Klebsiella pneumoniae in a hospital: Discovery of ST1869 clone and a novel hybrid plasmid" [Spectrum04195-25]. Those comments are all valuable and very helpful for revising and improving our paper, and are of great guiding significance to our research. All revisions have been highlighted in yellow in the marked-up manuscript.

Response to comments from Reviewer #2:

Comment 1: What is the rationale behind selecting only five clinical isolates from a single hospital? It limits generalizability.

Response: We thank the reviewer for this important concern. We fully acknowledge that five isolates from a single hospital limit generalizability. Accordingly, we have revised the manuscript as follows:

1. Title and scope: Changed from "epidemic trends in South China" to a local genomic characterization of the ST1869 clone and novel hybrid plasmid in our hospital.
2. Abstract and Conclusion: Removed statements implying regional outbreaks or broad epidemiological conclusions; rephrased to emphasize institutional genomic diversity.
3. Discussion: Explicitly stated this is a single-center study with small sample size (n=5, including three from one patient), and that regional prevalence requires multicenter validation.
4. Limitations: Added a clear statement that the small sample size limits extrapolation to broader populations.

The revised manuscript no longer claims generalizability beyond our institution, but rather provides baseline genomic data for future prospective studies. We believe these revisions adequately address the concern.

Comment 2: Shed some discussion on the presence of strains that showed susceptibility besides carrying resistance genes (blaNDM).

Response: We thank the reviewer for this valuable suggestion. We have added a paragraph in the

Discussion to address why bla_{NDM}-positive transconjugants (C23041N and C23071N) remained susceptible or showed low-level resistance to cefepime and ertapenem. We explain this from three perspectives: (i) plasmid adaptive evolution (structural variations reducing metabolic burden), (ii) regulatory differences affecting gene expression, and (iii) plasmid incompleteness and instability after transfer. We also note that resistance genes alone do not fully predict phenotypic outcomes, and integrated analysis of susceptibility testing, plasmid structure, and expression evidence is needed.

Comment 3: For strain 231-20. No transconjugants were obtained, besides detecting oriT sequence. What are the reasons?

Response: We thank the reviewer for this important question. In the revised manuscript, we have added a clear explanation in the Discussion section. Specifically, we state that the presence of an oriT sequence is necessary but not sufficient for conjugation; without a complete conjugative transfer system (including T4SS, T4CP, and relaxase), conjugation cannot initiate even when oriT is present. This directly addresses the apparent contradiction between the detection of oriT and the failure to obtain transconjugants from strain 231-20.

Additionally, adaptive evolution of plasmids in the host (e.g., truncations or deletions) may further reduce transfer efficiency, but the primary reason remains the incompleteness of the conjugation machinery.

Comment 4: Provide an improved method to analyze parameters such as SNA Calling thresholds, plasmid clustering, etc.

Response: We thank the reviewer for this suggestion. The revised Materials and Methods section now includes detailed parameters for both analyses: Snippy v4.6.0 was run with minimum sequencing depth ≥10×, base quality ≥20, and allele frequency ≥75% (20); Panaroo v1.3.2 was run in strict mode with a sequence identity threshold of ≥95% (23). These thresholds follow established standards for bacterial genomic surveillance and the developers' recommendations for accurate prokaryotic pan-genome construction, respectively.

Comment 5: Is there any evidence showed the single allele (phoE allele in ST1869 or its absence

in ST11) variant influence pathogenicity, virulence, environmental fitness or resistance?

Response: We thank the reviewer for this important point. We fully acknowledge that there is currently no direct evidence linking the *phoE*1/*phoE*9 allele difference to altered pathogenicity, virulence, fitness, or resistance. In the revised Discussion, we have explicitly stated that while *phoE* mutations have been implicated in carbapenem resistance, evidence remains insufficient to confirm whether the *phoE*1-to-*phoE*9 transition modulates these phenotypes (references (27–31)). We only suggest that the ST1869 clone warrants further surveillance given its close relatedness to local strain KC263, without claiming functional consequences attributable to this allele change. We have also noted that future isogenic allele-swap or competitive growth assays are needed to address this question, as added to the Discussion and Limitations.

Comment 6: Including patient therapeutic outcome will further strengthen the manuscript.

Response: We thank the reviewer for this suggestion. We agree that clinical outcomes would strengthen the study; however, as this was a retrospective genomic characterization using anonymized routine surveillance isolates, detailed treatment and prognostic data were unavailable under hospital ethical regulations. We have explicitly noted this limitation in the Limitations section.

Comment 7: Add Future direction, in terms of waste water surveillance or clinical surveillance.

Response: We thank the reviewer for this suggestion. Rather than adding a separate "Future directions" section, we have incorporated these perspectives into the existing manuscript structure to maintain logical flow. Specifically, we added a sentence at the end of the **Limitations** section stating: *"In addition, given recent reports of KN-CRKP in wastewater, future molecular monitoring that combines clinical and environmental specimens may further elucidate the genetic diversity of these plasmids."* This addresses the need for wastewater surveillance under a One Health framework, as well as prospective clinical surveillance. Furthermore, the **Conclusion** retains the recommendation for enhanced surveillance of the ST1869 clone and the novel IncI1/X3 plasmid, which aligns with the call for expanded multicenter monitoring. We believe these revisions appropriately integrate future directions without disrupting the manuscript's structure.

Comment 8: Add limitation of the study

Response: We thank the reviewer for this suggestion. We have added a dedicated “Limitations” section immediately after the Discussion and before the Conclusion, explicitly stating: (1) small sample size (*n*=5, including three from the same patient) limiting generalizability; (2) conjugation assays performed under single selective conditions without WGS of transconjugants; (3) unassessed long-term stability of the novel IncI1/X3 plasmid; and (4) unavailable clinical data due to anonymization. A call for future prospective, multicenter studies is also included.

Response to comments from Reviewer #3:

Major Comment 1: The bioinformatics analysis is relatively limited for a study making genomic epidemiology claims. The manuscript does not include a bacterial whole-genome phylogenetic analysis that is sufficiently informative for epidemiology. The chromosomal analysis appears limited to a small core-genome SNP comparison, while much of the study relies on MLST, plasmid typing, structural comparison, oriT prediction, and plasmid clustering. These analyses are useful descriptively, but they are not sufficient to support strong conclusions about transmission, outbreak structure, or regional dissemination.

Response: We thank the reviewer for this critical comment. We fully agree that the methods employed—core-genome SNP comparison, MLST, plasmid typing, and clustering—are descriptive and insufficient to support epidemiological conclusions regarding transmission or regional dissemination. Accordingly, we have fundamentally reframed the manuscript from a "genomic epidemiology" study to a "local genomic characterization" of KN-CRKP in our hospital. Under this revised scope: (i) core-genome SNP analysis is used solely to assess genetic relatedness (e.g., 3 SNPs between 231-20 and KC263), not to infer transmission chains; (ii) MLST and plasmid typing report clonal backgrounds and plasmid architectures found in our institution, not regional epidemic trends; and (iii) the 126 public genomes serve only as a comparative background to illustrate plasmid diversity. We have also added detailed bioinformatics parameters (SNP calling: depth ≥10×, quality ≥20, allele frequency ≥75%; Panaroo: strict mode, identity ≥95%) to enhance reproducibility. The revised manuscript no longer claims regional outbreaks or transmission pathways, and focuses strictly on describing the genomic features, resistance

mechanisms, and plasmid diversity of our isolates. We believe these revisions adequately address the reviewer's concern.

Major Comment 2: The epidemiological claims are too strong for the data presented. The manuscript repeatedly refers to regional outbreaks, transmission pathways, frequent plasmid transmission, and regional prevalence, but the primary dataset consists of only five local isolates collected over a short period, three from the same patient. The manuscript would be stronger if framed primarily as a genomic characterization study with comparative context rather than as definitive evidence of epidemic trends in Southern China.

Response: We thank the reviewer for this critical comment. We fully agree that five isolates (including three from the same patient) cannot support regional epidemiological conclusions. We have therefore fundamentally reframed the manuscript from a "regional epidemic trend analysis" to a "genomic characterization of KN-CRKP in our hospital." Specifically: (1) the title no longer references epidemic trends in South China; (2) the Abstract and Conclusion have been revised to remove all statements implying regional outbreaks, transmission pathways, or prevalence; (3) the Introduction has been toned down to emphasize local data gaps; (4) the Discussion no longer uses terms such as "outbreak" or "regional dissemination," and explicitly states that the 126 public genomes serve solely as comparative genomic background; and (5) the Limitations section clearly notes the small sample size (n = 5, with three from the same patient). The revised manuscript now serves purely as a detailed genomic characterization of KN-CRKP in our institution, without any regional epidemiological claims. We believe these revisions fully address the reviewer's concern.

Major Comment 3: The sequencing and assembly strategy is unclear. The methods suggest that only representative isolates underwent long-read sequencing, while the results specifically identify 230-25 and 231-20 as long-read sequenced, yet the data availability section implies complete long-read assemblies for all five isolates. This needs to be clarified explicitly.

Response: We thank the reviewer for pointing out this lack of clarity. We have explicitly clarified in the **Materials and Methods** that all five isolates underwent Illumina short-read sequencing, while only representative strains 230-25 (ST11) and 231-20 (ST1869) received additional Nanopore long-read sequencing. The **Data availability** section has also been revised to clearly

distinguish the two complete hybrid assemblies (SAMN53171962 and SAMN53172020) from the three Illumina-only draft assemblies (SAMN53628860, SAMN53628922, and SAMN53629510). These changes eliminate any implication that all five isolates have complete long-read assemblies.

Major Comment 4: Several conclusions about plasmid dissemination and circulation go beyond the evidence presented. The manuscript often moves from plasmid similarity, oriT prediction, or limited conjugation results to stronger claims about dissemination and regional circulation. These should be presented more cautiously as hypotheses rather than direct conclusions.

Response: We thank the reviewer for this important comment. We fully agree that the original manuscript drew overly strong conclusions from limited evidence. We have systematically revised the text to present these findings as hypotheses rather than definitive conclusions. Deterministic terms such as "outbreak," "transmission pathway," and "regional dissemination" have been replaced with cautious language ("potentially," "suggestive," "warrants surveillance"). We now clarify that plasmid similarity, oriT prediction, and conjugation results indicate potential transferability rather than proven transmission. The Conclusion has been revised to recommend targeted surveillance of the ST1869 clone, IncI1/X3 plasmids, and novel transposon modules, removing claims of multi-site outbreaks or inter-clone transmission. We have also explicitly stated in the Limitations that the 126 public genomes serve solely as comparative genomic background and do not support independent epidemiological inference. The revised manuscript no longer treats these data as direct evidence of dissemination, but rather as a basis for surveillance hypotheses. We believe these revisions adequately address the reviewer's concern.

Major Comment 5: The local epidemiological metadata are too limited to support transmission-level interpretation. Apart from specimen source and the fact that three isolates came from the same patient, there is little clinical or hospital context presented. Without clearer metadata, the genomic findings cannot be interpreted robustly in an epidemiological framework.

Response: We thank the reviewer for this comment. We fully agree that limited clinical metadata preclude robust epidemiological interpretation. Accordingly, we have reframed the study as a pure genomic characterization and explicitly state in the **Materials and Methods**, **Results**, and **Limitations** that detailed hospital metadata (ward, department, treatment regimens, outcomes)

were unavailable due to record anonymization. All epidemiological inference terms (e.g., “outbreak,” “transmission chain,” “regional dissemination”) have been removed throughout the manuscript. The revised version now reports only the resistance phenotypes, plasmid structures, and genetic contexts of the five isolates without any transmission-level claims.

Major Comment 6: The comparative genomics workflow is not sufficiently reproducible. The manuscript states that public genomes were retrieved and filtered to 126 high-quality genomes, but the search strategy, inclusion criteria, accession list, and definition of invalid entries are not described clearly enough.

Response: We thank the reviewer for this comment. We have added detailed retrieval parameters to the **Materials and Methods**: the NCBI Pathogen Detection database was queried using "*bla*$_{\mathrm{KPC}}$" and "*bla*$_{\mathrm{NDM}}$" (species: *K. pneumoniae*) on 20 June 2025. Inclusion criteria were: (1) complete genome or circular plasmid indicated; (2) draft genomes with contig ≤200 and N50 ≥50,000 bp; (3) exclusion of chromosome-only assemblies. Entries marked as "suppressed" or "surveillance assembly" by NCBI RefSeq were excluded. All 126 included isolates are listed in **Table S2** with accession numbers, STs, and metadata, ensuring full reproducibility.

Minor Comment : The manuscript would benefit from substantial language editing, as there are multiple formatting and writing issues, including inconsistent headings and typographical errors. Some speculative interpretations, including genotype-phenotype explanations and broader public health implications, should also be toned down.

Response: We thank the reviewer for these suggestions. We have thoroughly revised the manuscript for language, formatting, and tone. Specifically: (1) heading styles have been unified and typographical errors corrected; (2) speculative genotype-phenotype explanations are now explicitly framed as hypotheses requiring further experimental validation; (3) public health implications have been toned down to our hospital context only, with all regional epidemiological claims removed.

Re: Spectrum04195-25R1 (Genomic characterization of KPC-2 and NDM coproducing carbapenem-resistant Klebsiella pneumoniae in a hospital: Discovery of ST1869 clone and a novel hybrid plasmid)

Dear Dr. Shihan Zeng:

Your manuscript has been accepted, and I am forwarding it to the ASM production staff for publication. Your paper will first be checked to make sure all elements meet the technical requirements. ASM staff will contact you if anything needs to be revised before copyediting and production can begin. Otherwise, you will be notified when your proofs are ready to be viewed.

Data Availability: ASM policy requires that data be available to the public upon online posting of the article, so please verify all links to sequence records, if present, and make sure that each number retrieves the full record of the data. If a new accession number is not linked or a link is broken, provide production staff with the correct URL for the record. If the accession numbers for new data are not publicly accessible before the expected online posting of the article, publication may be delayed; please contact ASM production staff immediately with the expected release date.

Publication Fees: For information on publication fees and which article types have charges, please visit our website. We have partnered with Copyright Clearance Center (CCC) to collect author charges. If fees apply to your paper, you will receive a message from no-reply@copyright.com with further instructions. For questions related to paying charges through RightsLink, please contact CCC at ASM_Support@copyright.com or toll free at +1-877-622-5543. CCC makes every attempt to respond to all emails within 24 hours.

ASM Membership: Corresponding authors may join or renew ASM membership to obtain discounts on publication fees. Need to upgrade your membership level? Please contact Customer Service at Service@asmusa.org.

Embargo Policy: A press release may be issued as soon as the manuscript is posted on the Spectrum Latest Articles webpage. The corresponding author will receive an email with the subject line "ASM Journals Author Services Notification" when the article is available online.

The ASM Journals program strives for constant improvement in our submission and publication process. Please tell us how we can improve your experience by taking this quick Author Survey.

Thank you for submitting your paper to Spectrum.

Sincerely,
Matt Bawn
Editor
Microbiology Spectrum
