# Supplementary material for: Genomic characterization of KPC-2 and NDM coproducing carbapenem-resistant Klebsiella pneumoniae in a hospital: discovery of ST1869 clone and a novel hybrid plasmid
Source: Microbiol Spectr. 2026 Aug 13;14(9):e04195-25. doi: 10.1128/spectrum.04195-25 (PMC13532327; doi:10.1128/spectrum.04195-25)
Supplement: Supplementary file 2 [file spectrum.04195-25-s0001.docx]

# Supplementary Figures

Figure S1. Comparison of KPC-2- and NDM-carrying plasmids with related plasmids. (A) Comparison of p23025-KPC2, p23120-KPC2 and other similar plasmids (B) Comparison of p23025-NDM1, p23120-NDM5 and other similar plasmids. Open reading frames are indicated by arrows. Red, resistance genes; green, mobile genetic elements; blue, functional protein genes; grey, other genes. Open reading frames are indicated by arrows. Red, resistance genes; green, mobile genetic elements; blue, functional protein genes; purple, conjugal transfer protein; grey, other genes.

Figure S2 Representative genetic environments of *bla*_KPC-2_ in KPC-NDM (Type I–IV). (A) KPC-type-I: *hp-klcA-hp-KorC-ΔISKpn6-bla*_KPC-2_*-ISKpn27-tnpR*; (B) KPC-type-II: *ISKpn6-bla*_KPC-2_*-istB-istA-tnpA-tnpR*; (C) KPC-type-III: *IS21-Tn3-IS26-ΔISKpn26-bla*_KPC-2_*-ISKpn27-recombinase-IS26*; (D) KPC-type-IV: *IS26-ISKpn6-bla*_KPC-2_*-ISKpn27-IS26-IS26-transposase-QnrS1*. Open reading frames are indicated by arrows. Red, resistance genes; green, mobile genetic elements; blue, functional protein genes; grey, other genes.

Figure S3 KN-CRKP *bla*_NDM_ Genetic structure. Open reading frames are indicated by arrows. Red, resistance genes; green, mobile genetic elements; blue, functional protein genes; grey, other genes.

Figure S4 Representative genetic environments of *bla*_NDM_ in KN-CRKP (Type I–IV). (A) NDM-type-I: *bla*_NDM-1_-*ble-trpF-dsbD-cutA-groS-groL-IS91*; (B) NDM-type-II: *bla*_NDM-1_~-*ble-trpF-dsbD*; (C) NDM-type-III: *bla*_NDM-1_-*ble-trpF*; (D) NDM-type-IV: *ISKpn19-IS3000- bla*_NDM-1_*-ISKpn19-Rec-ORF3-Tn3-IS26*. Open reading frames are indicated by arrows. Red, resistance genes; green, mobile genetic elements; blue, functional protein genes; grey, other genes.

Figure S5 Comparison of p23025-KPC2, p23120-KPC2 and KN-CRKP *bla*_KPC_ positive plasmids in South China. Open reading frames are indicated by arrows. Red, resistance genes; green, mobile genetic elements; blue, functional protein genes; purple, conjugal transfer protein; grey, other genes.

Figure S6 Comparison of p23025-NDM1, p23120-NDM5 and KN-CRKP *bla*_NDM_ positive plasmids in South China. Open reading frames are indicated by arrows. Red, resistance genes; green, mobile genetic elements; blue, functional protein genes; purple, conjugal transfer protein; grey, other genes.
