# Supplementary material for: Genomic characterization of KPC-2 and NDM coproducing carbapenem-resistant Klebsiella pneumoniae in a hospital: discovery of ST1869 clone and a novel hybrid plasmid
Source: Microbiol Spectr. 2026 Aug 13;14(9):e04195-25. doi: 10.1128/spectrum.04195-25 (PMC13532327; doi:10.1128/spectrum.04195-25)
Supplement: Supplementary file 3 [file spectrum.04195-25-s0008.docx]

# Supplement Tables

[Table S1. Primers used for PCR detection of *bla*_NDM_ and *bla*_KPC_ genes	2](#_Toc231030451)

[Table S2. Microbiological characteristics of 126 KN-CRKP	3](#_Toc231030452)

[Table S3 plasmids carried by the isolate and the drug resistance gene located on the plasmid	17](#_Toc231030453)

Table S1. Primers used for PCR detection of *bla*_NDM_ and *bla*_KPC_ genes

| Primer Name | Sequence（5'→3'） | Target gene | Product length (bp^2^) |
| --- | --- | --- | --- |
| *bla*_NDM_ - F | TGGACCGATGACCAGACC^a^ | *bla*_NDM_ | 443 |
| *bla*_NDM_ - R | AACGCCGCACCAAACG |  |  |
| *bla*_KPC_ - F | GGAACCATTCGCTAAACTCG | *bla*_KPC_ | 782 |
| *bla*_KPC_ - R | TGACGCCCAATCCCTC |  |  |

1. Initial denaturation at 94°C for 5 min; 35 cycles of denaturation at 94°C for 30 s, annealing at 53°C for 45 s, and extension at 72°C for 1 min; followed by a final extension at 72°C for 5 min.

2. base pairs

Table S2. Microbiological characteristics of 126 KN-CRKP

| Assembly | Specimen | Province | NDM Gene | KPC Gene | MLST^1^ | NDM Inc^2^ | KPC Inc | NDM-type | KPC-type |
| --- | --- | --- | --- | --- | --- | --- | --- | --- | --- |
| GCA_002944845.1 | tissue sample | Jiangxi | *bla*_NDM-1_ | *bla*_KPC-2_ | 86 | IncN | IncFII | I | I |
| GCA_010183605.1 | sputum | Shandong | *bla*_NDM-1_ | *bla*_KPC-2_ | 15 | IncN2 | IncFII | III | I |
| GCA_010183625.1 | sputum | Shandong | *bla*_NDM-1_ | *bla*_KPC-2_ | 15 | IncN2 | IncFII | III | I |
| GCA_010183645.1 | pus | Beijing | *bla*_NDM-1_ | *bla*_KPC-2_ | 11 | IncX3 | IncFII/FR | I | I |
| GCA_010183685.1 | urine | Hubei | *bla*_NDM-1_ | *bla*_KPC-2_ | 11 | IncN | IncFII/FR | I | I |
| GCA_010183705.1 | intra-abdominal fluid | Shandong | *bla*_NDM-1_ | *bla*_KPC-2_ | 3493 | IncFIB/HI5B | IncFIA/FII | I | I |
| GCA_014961485.1 | blood | Henan | *bla*_NDM-1_ | *bla*_KPC-2_ | 2667 | IncFIB/HI5B/Q1 | IncFII | II | I |
| GCA_015238555.1 | sputum | Henan | *bla*_NDM-1_ | *bla*_KPC-2_ | 2667 | IncFIB/HI5B/Q1 | IncFII | II | I |
| GCA_016812055.1 | tissue sample | Jiangsu | *bla*_NDM-5_ | *bla*_KPC-2_ | 11 | IncB/O/K/Z | IncFII/FR | I | I |
| GCA_018274525 | intestine | others countries | *bla*_NDM-1_ | *bla*_KPC-2_ | 39 | IncA/C2 | IncFIB/FII | I | II |
| GCA_018283575.2 | intestine | others countries | *bla*_NDM-1_ | *bla*_KPC-2_ | 39 | IncA/C2 | IncFIB/FII | I | II |
| GCA_018314145.1 | intra-abdominal fluid | Henan | *bla*_NDM-5_ | *bla*_KPC-2_ | 11 | others | IncFII/N/R | I | I |
| GCA_018383785.2 | tracheal aspirate | others countries | *bla*_NDM-1_ | *bla*_KPC-2_ | 39 | IncA/C2 | IncFIB/FII | I | II |
| GCA_018682475.1 | pancreas | Jiangsu | *bla*_NDM-1_/ *bla*_NDM-5_ | *bla*_KPC-2_ | 11 | IncFII/N/R / Chromosome | IncFII/N/R | II | I |
| GCA_018682505.1 | pancreas | Jiangsu | *bla*_NDM-5_ | *bla*_KPC-2_ | 11 | IncFII/N/R | IncFII/N/R | II | I |
| GCA_018682545.1 | pancreas | Jiangsu | *bla*_NDM-5_ | *bla*_KPC-2_ | 11 | IncFII/N/R | IncFII/N/R | II | I |
| GCA_019444195.1 | sputum | Hubei | *bla*_NDM-1_ | *bla*_KPC-2_ | 11 | IncFII | IncFII/R | I | I |
| GCA_020520225.1 | unknown | Hubei | *bla*_NDM-5_ | *bla*_KPC-2_ | 11 | IncI1 | IncFII/R | II | I |
| GCA_020535145.1 | blood | Henan | *bla*_NDM-1_ | *bla*_KPC-2_ | 2667 | IncFIB/HI5B/Q1 | IncFII | II | I |
| GCA_021397585.1 | Cerebral spinal fluid | Zhejiang | *bla*_NDM-1_ | *bla*_KPC-2_ | 15 | IncX3 | IncFII | I | I |
| GCA_021398155.1 | sputum | Shandong | *bla*_NDM-1_ | *bla*_KPC-2_ | 11 | IncA/C2 | IncFII/R | I | I |
| GCA_022059905.1 | unknown | Zhejiang | *bla*_NDM-1_ | *bla*_KPC-2_ | 11 | IncFIB/HI5B | IncN/U | I | I |
| GCA_022453645.1 | urine | Zhejiang | *bla*_NDM-1_ | *bla*_KPC-2_ | 11 | IncFIB/HI5B | IncN/U | I | I |
| GCA_022592735.1 | blood | Sichuan | *bla*_NDM-1_ | *bla*_KPC-2_ | 464 | IncX3 | IncFIB/HI5 | I | I |
| GCA_022649725.1 | unknown | Sichuan | *bla*_NDM-5_ | *bla*_KPC-2_ | 11 | IncX3 | IncFII/FR | II | III |
| GCA_022748955.1 | tracheal aspirate | others countries | *bla*_NDM-1_ | *bla*_KPC-2_ | 23 | IncFIB/HI1B | IncFIB/FII | I | II |
| **GCA_024496165.1**^3^ | **sputum** | **Guangdong** | ***bla*_NDM-1_** | ***bla*_KPC-2_** | **11** | **IncX3** | **IncFII/R** | **I** | **I** |
| GCA_024762135.1 | blood | Chongqing | *bla*_NDM-5_ | *bla*_KPC-2_ | 11 | IncX3 | IncFII/R | I | I |
| GCA_025673225.1 | unknown | Shanghai | *bla*_NDM-5_ | *bla*_KPC-2_ | 15 | IncFII | IncFII | II | I |
| GCA_025674335.1 | unknown | Shanghai | *bla*_NDM-5_ | *bla*_KPC-2_ | 11 | IncFII / Chromosome / unknown | IncFII/R | II | I |
| GCA_025677745.1 | blood | Beijing | *bla*_NDM-5_ | *bla*_KPC-2_ | 11 | IncX3 | IncFII/R | I | I |
| GCA_025677765.1 | blood | Beijing | *bla*_NDM-5_ | *bla*_KPC-2_ | 11 | IncX3 | IncFII/R | I | I |
| GCA_025946965.1 | stool | Zhejiang | *bla*_NDM-1_ | *bla*_KPC-2_ | 15 | IncFIB/FII | others | I | I |
| GCA_026167905.1 | blood | Hebei | *bla*_NDM-1_ | *bla*_KPC-2_ | 1414 | Chromosome | Chromosome | I | I |
| GCA_026223255.1 | unknown | others countries | *bla*_NDM-7_ | *bla*_KPC-2_ | 11 | IncX3 | IncFII | I | II |
| GCA_028220555.1 | sputum | Hubei | *bla*_NDM-1_ | *bla*_KPC-2_ | 1049 | IncL/M | IncL/M | I | IV |
| GCA_028527255.1 | unknown | Beijing | *bla*_NDM-1_ | *bla*_KPC-2_ | 22 | IncFIB/FII | IncFII | I | I |
| GCA_028527285.1 | unknown | Beijing | *bla*_NDM-1_ | *bla*_KPC-2_ | 22 | IncFIB/FII | IncFII | I | I |
| GCA_028527305.1 | unknown | Beijing | *bla*_NDM-1_ | *bla*_KPC-2_ | 22 | IncFIB/FII | IncFII | I | I |
| GCA_028527345.1 | unknown | Beijing | *bla*_NDM-1_ | *bla*_KPC-2_ | 22 | IncFIB/FII | IncFII | I | I |
| GCA_028527365.1 | unknown | Beijing | *bla*_NDM-1_ | *bla*_KPC-2_ | 22 | IncFIB/FII | IncFII | I | I |
| GCA_028527385.1 | unknown | Beijing | *bla*_NDM-1_ | *bla*_KPC-2_ | 22 | IncFIB/FII | IncFII | I | I |
| GCA_028527395.1 | unknown | Beijing | *bla*_NDM-1_ | *bla*_KPC-2_ | 22 | IncFIB/FII | IncFII | I | I |
| GCA_028527425.1 | unknown | Beijing | *bla*_NDM-1_ | *bla*_KPC-2_ | 22 | IncFIB/FII | IncFII | I | I |
| GCA_028527445.1 | unknown | Beijing | *bla*_NDM-1_ | *bla*_KPC-2_ | 22 | IncFIB/FII | IncFII | I | I |
| GCA_028527465.1 | unknown | Beijing | *bla*_NDM-1_ | *bla*_KPC-2_ | 22 | IncFIB/FII | IncFII | I | I |
| GCA_028527475.1 | unknown | Beijing | *bla*_NDM-1_ | *bla*_KPC-2_ | 22 | IncFIB/FII | IncFII | I | I |
| GCA_028527485.1 | unknown | Beijing | *bla*_NDM-1_ | *bla*_KPC-2_ | 22 | IncFIB/FII | IncFII | I | I |
| GCA_029223325.1 | sputum | others countries | *bla*_NDM-1_ | *bla*_KPC-3_ | 512 | IncFIB/HI1B | IncX3 | II | I |
| GCA_029458035.1 | tissue sample | others countries | *bla*_NDM-1_ | *bla*_KPC-2_ | 225 | IncFIB/FII | Chromosome | II | II |
| GCA_029623595.1 | tracheal aspirate | others countries | *bla*_NDM-1_ | *bla*_KPC-3_ | 307 | IncFII | IncX3 | II | II |
| GCA_030388425.1 | blood | Zhejiang | *bla*_NDM-1_ | *bla*_KPC-2_ | 11 | IncFIB/HI1B/X3 | IncFII/R | I | I |
| GCA_030552935.1 | sputum | Zhejiang | *bla*_NDM-5_ | *bla*_KPC-2_ | 11 | IncFII | others | II | I |
| GCA_031624135.1 | sputum | Zhejiang | *bla*_NDM-5_ | *bla*_KPC-2_ | 11 | IncHI2A/HI2+RepA | IncFII/R | II | I |
| GCA_031624185.1 | unknown | Zhejiang | *bla*_NDM-5_ | *bla*_KPC-2_ | 11 | others | IncFII/R | II | I |
| GCA_031676375.1 | sputum | Henan | *bla*_NDM-5_ | *bla*_KPC-2_ | 11 | IncFIA/FIB/FIC | IncFII/R | II | I |
| GCA_033807335.1 | urine | Hubei | *bla*_NDM-1_ | *bla*_KPC-2_ | 11 | IncA/C2 | IncFII/R | I | III |
| GCA_033807355.1 | urine | Hubei | *bla*_NDM-1_ | *bla*_KPC-2_ | 11 | IncN | IncFII/R | I | I |
| GCA_033807375.1 | urine | Hubei | *bla*_NDM-1_ | *bla*_KPC-2_ | 11 | IncN | IncFII/R | I | III |
| GCA_033807395.1 | sputum | Hubei | *bla*_NDM-1_ | *bla*_KPC-2_ | 1049 | IncL/M | IncL/M | IV | IV |
| GCA_033807415.1 | blood | Hubei | *bla*_NDM-1_ | *bla*_KPC-2_ | 1049 | IncL/M | IncL/M | IV | IV |
| GCA_033807435.1 | sputum | Hubei | *bla*_NDM-1_ | *bla*_KPC-2_ | 1049 | IncL/M | IncL/M | IV | IV |
| GCA_033807455.1 | sputum | Hubei | *bla*_NDM-1_ | *bla*_KPC-2_ | 1049 | IncL/M | IncL/M | IV | IV |
| GCA_035201625.1 | intra-abdominal fluid | Zhejiang | *bla*_NDM-5_ | *bla*_KPC-2_ | 11 | IncFII/N/R | IncFII/N/R | II | I |
| GCA_036451995.1 | blood | others countries | *bla*_NDM-1_ | *bla*_KPC-3_ | 409 | IncA/C2 | IncA/C2_1 | III | II |
| GCA_036452175.1 | blood | others countries | *bla*_NDM-1_ | *bla*_KPC-3_ | 3515 | IncX3 | IncN | I | II |
| GCA_036884315.1 | sputum | Jiangsu | *bla*_NDM-5_ | *bla*_KPC-2_ | 11 | IncFII/N/R | IncFII/N/R | II | I |
| GCA_037023225.1 | sputum | Shandong | *bla*_NDM-1_ | *bla*_KPC-2_ | 11 | IncA/C2 | IncFII/R | II | I |
| GCA_040784875.1 | sputum | Hunan | *bla*_NDM-1_ | *bla*_KPC-2_ | 11 | IncN | IncFII/R | I | I |
| GCA_040894335.1 | tracheal aspirate | others countries | *bla*_NDM-1_ | *bla*_KPC-2_ | 258 | IncA/C2 | IncFIB/FII | I | II |
| GCA_041004885.1 | secretion | Yunnan | *bla*_NDM-1_ | *bla*_KPC-2_ | 11 | IncFIB/FII/N2 | IncFIB/FII/N2 | III | others |
| GCA_042134625.2 | blood | Beijing | *bla*_NDM-13_ | *bla*_KPC-2_ | 11 | IncI1 | IncFII/R | III | I |
| GCA_042134645.2 | blood | Beijing | *bla*_NDM-13_ | *bla*_KPC-2_ | 11 | IncI1 | IncFII/R | III | I |
| GCA_042607805.1 | unknown | others countries | *bla*_NDM-1_ | *bla*_KPC-2_ | 11 | IncFIB/HI1B | IncFIB/N | I | II |
| **GCA_043950135.1** | **sputum** | **Guangdong** | ***bla*_NDM-5_** | ***bla*_KPC-2_** | **11** | **IncX3** | **IncFII/R** | **I** | **I** |
| GCA_045069655.2 | tissue sample | others countries | *bla*_NDM-5_ | *bla*_KPC-3_ | 147 | IncHI1B | IncFII | II | II |
| GCA_045222865.1 | blood | others countries | *bla*_NDM-7_ | *bla*_KPC-2_ | 45 | IncX3 | IncFIB/FII | II | II |
| GCA_046118685.1 | unknown | Shanghai | *bla*_NDM-1_ | *bla*_KPC-2_ | 11 | IncFIB/FII | IncFII/R | III | I |
| GCA_046368075.1 | urine | Beijing | *bla*_NDM-1_ | *bla*_KPC-2_ | 22 | IncFIB/FII | IncFII | I | I |
| GCA_046368105.1 | Balf | Beijing | *bla*_NDM-1_ | *bla*_KPC-2_ | 11 | IncX3 | IncFII/R | I | III |
| GCA_046368125.1 | sputum | Beijing | *bla*_NDM-1_ | *bla*_KPC-2_ | 11 | IncX3 | IncFII/R | I | III |
| GCA_046368265.1 | Balf | Hunan | *bla*_NDM-1_ | *bla*_KPC-2_ | 11 | IncFII | IncFII/R | I | I |
| GCA_046543535.1 | Balf | others countries | *bla*_NDM-1_ | *bla*_KPC-2_ | 307 | IncA/C2 | IncFIB/HI1B_1 | I | II |
| GCA_046543595.1 | stool | others countries | *bla*_NDM-1_ | *bla*_KPC-3_ | 307 | IncA/C2 | IncR | I | II |
| GCA_046543665.1 | catheter | others countries | *bla*_NDM-1_ | *bla*_KPC-2_ | 307 | IncA/C2 | IncL/M | I | I |
| GCA_047456365.1 | stool | others countries | *bla*_NDM-1_ | *bla*_KPC-2_ | 23 | Chromosome | Chromosome | I | others |
| GCA_047783025.1 | sputum | Zhejiang | *bla*_NDM-1_ | *bla*_KPC-2_ | 1545 | IncFIB/HI5B | IncFII | I | I |
| GCA_047783035.1 | bile | Zhejiang | *bla*_NDM-1_ | *bla*_KPC-2_ | 1545 | IncFIB/HI5B | IncFII | I | I |
| GCA_047783045.1 | sputum | Zhejiang | *bla*_NDM-1_ | *bla*_KPC-2_ | 1545 | IncU | IncFII | I | I |
| GCA_047783055.1 | sputum | Zhejiang | *bla*_NDM-1_ | *bla*_KPC-2_ | 1545 | IncU | IncFII | I | I |
| GCA_047783065.1 | sputum | Zhejiang | *bla*_NDM-1_ | *bla*_KPC-2_ | 1545 | IncU | IncFII | I | III |
| GCA_047783075.1 | sputum | Zhejiang | *bla*_NDM-1_ | *bla*_KPC-2_ | 1545 | IncU | IncFII | I | III |
| GCA_047783085.1 | unknown | Zhejiang | *bla*_NDM-1_ | *bla*_KPC-2_ | 1545 | IncU | IncFII | II | III |
| **GCA_047948275.1** | **sputum** | **Fujian** | ***bla*_NDM-1_** | ***bla*_KPC-2_** | **11** | **IncFII/R** | **IncFII/R** | **I** | **III** |
| **GCA_048319915.1** | **sputum** | **Guangdong** | ***bla*_NDM-5_** | ***bla*_KPC-2_** | **15** | **IncFIB/X4** | **Chromosome** | **II** | **III** |
| **GCA_048319935.1** | **secretion** | **Guangdong** | ***bla*_NDM-1_** | ***bla*_KPC-2_** | **11** | **IncX3** | **IncFII/R** | **I** | **I** |
| **GCA_048319945.1** | **secretion** | **Guangdong** | ***bla*_NDM-5_** | ***bla*_KPC-2_** | **1869** | **IncI1/X3** | **IncFII/R** | **II** | **I** |
| GCA_048320185.1 | secretion | Henan | *bla*_NDM-5_ | *bla*_KPC-2_ | 11 | IncX3 | IncFII/R | II | I |
| GCA_048568885.1 | secretion | Shanghai | *bla*_NDM-1_ | *bla*_KPC-2_ | 11 | IncX3 | IncFII/R | I | I |
| GCA_049348255.1 | unknown | unknown | *bla*_NDM-1_ | *bla*_KPC-2_ | 45 | IncX3 | repB | II | I |
| GCA_049349765.1 | unknown | unknown | *bla*_NDM-1_ | *bla*_KPC-14_ | 11 | IncX3 | IncFII | I | I |
| GCA_049354605.1 | urine | others countries | *bla*_NDM-5_ | *bla*_KPC-3_ | 6260 | Chromosome | IncX3 | I | II |
| GCA_049354615.1 | urine | others countries | *bla*_NDM-5_ | *bla*_KPC-3_ | 6260 | Chromosome | IncX3 | I | II |
| GCA_049554945.1 | unknown | unknown | *bla*_NDM-1_ | *bla*_KPC-2_ | 11 | IncFIB/FII | IncFII/R | I | I |
| GCA_050425265.1 | intra-abdominal fluid | Jiangxi | *bla*_NDM-1_ | *bla*_KPC-2_ | 11 | IncX3 | IncFII/R | I | III |
| GCA_050425875.1 | intra-abdominal fluid | Jiangxi | *bla*_NDM-1_ | *bla*_KPC-2_ | 11 | IncX3 | IncFII/R | I | III |
| GCA_050507665.1 | sewage | Sichuan | *bla*_NDM-1_ | *bla*_KPC-2_ | 34 | IncU | IncFII | I | III |
| **GCA_050562205.1** | **unknown** | **Fujian** | ***bla*_NDM-5_** | ***bla*_KPC-2_** | **11** | **IncI1** | **IncFII** | **II** | **III** |
| GCA_050808305.1 | sputum | Beijing | *bla*_NDM-1_ | *bla*_KPC-2_ | 11 | IncX3 | IncFII/R | I | III |
| GCA_050808315.1 | balf | Beijing | *bla*_NDM-1_ | *bla*_KPC-2_ | 11 | IncX3 | IncFII/R | I | III |
| GCA_050809045.1 | urine | Beijing | *bla*_NDM-1_ | *bla*_KPC-2_ | 22 | IncFIB/FII | IncFII | I | I |
| GCA_050809065.1 | balf | Beijing | *bla*_NDM-1_ | *bla*_KPC-2_ | 11 | IncA/C2 | IncI1 | I | I |
| GCA_050809825.1 | blood | Beijing | *bla*_NDM-1_ | *bla*_KPC-2_ | 11 | IncA/C2 | IncI1 | I | I |
| GCA_050809835.1 | Secretion | Beijing | *bla*_NDM-5_ | *bla*_KPC-2_ | 11 | IncX3 | IncFII/R | I | I |
| GCA_050810525.1 | balf | Beijing | *bla*_NDM-5_ | *bla*_KPC-2_ | 11 | IncFIA/FII | IncFII | I | III |
| GCA_050810535.1 | balf | Beijing | *bla*_NDM-1_ | *bla*_KPC-33_ | 11 | IncFIB/FII | IncFII/R | I | I |
| GCA_050811135.1 | sputum | Beijing | *bla*_NDM-13_ | *bla*_KPC-2_ | 11 | IncI1 | IncFII/R | III | I |
| GCA_050811155.1 | blood | Beijing | *bla*_NDM-13_ | *bla*_KPC-2_ | 11 | IncI1 | IncFII/R | III | I |
| GCA_050811865.1 | balf | Beijing | *bla*_NDM-1_ | *bla*_KPC-33_ | 11 | IncFIB/FII | IncFII/R | I | I |
| GCA_050811875.1 | urine | Beijing | *bla*_NDM-13_ | *bla*_KPC-2_ | 11 | IncI1 | IncFII/R | III | I |
| GCA_050812445.1 | balf | Beijing | *bla*_NDM-1_ | *bla*_KPC-2_ | 11 | IncN | IncFII/R | I | I |
| **GCA_050812455.1** | **Secretion** | **Guangxi** | ***bla*_NDM-1_** | ***bla*_KPC-2_** | **15** | **IncX3** | **IncN** | **I** | **I** |
| GCA_050813175.1 | unknown | Henan | *bla*_NDM-5_ | *bla*_KPC-2_ | 11 | IncX3 | IncFII | II | III |
| GCA_050871095.1 | blood | others countries | *bla*_NDM-5_ | *bla*_KPC-2_ | 307 | IncFIB/HI1B | IncFIB/FII | II | II |
| GCA_050871105.1 | tracheal aspirate | others countries | *bla*_NDM-5_ | *bla*_KPC-2_ | 307 | IncFIB/HI1B | IncL/M | II | I |
| GCA_050871115.1 | blood | others countries | *bla*_NDM-1_ | *bla*_KPC-3_ | 307 | IncA/C2 | IncR | I | II |

1. Multilocus sequence typing

2. incompatibility group

3. Bold indicates KN-CRKP from southern China

Table S3 plasmids carried by the isolate and the drug resistance gene located on the plasmid

| Plasmid | Length (bp^1^) | Plasmid replicon | Resistance genes |
| --- | --- | --- | --- |
| p23025-KPC2 | 55730 | IncFII | *bla*_KPC-2_, *bla*_TEM-1_, *rmtB*, *bla*_SHV-12_ |
| p23025-NDM1 | 85718 | IncFII | *bla*_NDM-1_, *ble*_MBL_, *bla*_TEM_, *rmtB* |
| p23025-contig2 | 189700 | IncFIB/FII | *bla*_LAP-2_, *qnrS1*, *sul2*, *tet(A)* |
| p23120-KPC2 | 118646 | IncFII/R | *bla*_KPC-2_, *bla*_CTX-M-65_, *rmtB*, *bla*_TEM-1_ |
| p23120-NDM5 | 64902 | IncI1/X3 | *bla*_NDM-5_, *ble*_MBL_, *erm(B)*, *bla*_CMY-42_ |
| p23120-contig4 | 86200 | IncFII | *bla*_LAP-2_, *qnrS1*, *sul2*, *tet(A), dfrA14* |

1. base pairs
